# The Effect of Different Ester Chain Modifications of Two Guaianolides for Inhibition of Colorectal Cancer Cell Growth

**DOI:** 10.3390/molecules26185481

**Published:** 2021-09-09

**Authors:** Lamis Al Aaraj, Berthe Hayar, Zaynab Jaber, Walid Saad, Najat A. Saliba, Nadine Darwiche, Tarek Ghaddar

**Affiliations:** 1 AUB Nature Conservation Center, American University of Beirut, Beirut P.O. Box 11-0236, Lebanon; lamis.agha86@gmail.com (L.A.A.); bh48@aub.edu.lb (B.H.); Zjaber90@gmail.com (Z.J.); ws20@aub.edu.lb (W.S.); ns30@aub.edu.lb (N.A.S.); 2 Department of Chemistry, American University of Beirut, Beirut P.O. Box 11-0236, Lebanon; 3 Department of Biochemistry and Molecular Genetics, American University of Beirut, Beirut P.O. Box 11-0236, Lebanon; 4 Department of Chemical Engineering, American University of Beirut, Beirut P.O. Box 11-0236, Lebanon

**Keywords:** guaianolides, sesquiterpene lactones, ester derivatives, cell growth activity, colorectal cancer, p53

## Abstract

Several sesquiterpene lactones (STLs) have been tested as lead drugs in cancer clinical trials. Salograviolide-**A** (Sal-**A)** and salograviolide-**B** (Sal-**B)** are two STLs that have been isolated from *Centaurea ainetensis,* an indigenous medicinal plant of the Middle Eastern region. The parent compounds Sal-**A** and Sal-**B** were modified and successfully prepared into eight novel guaianolide-type STLs (compounds **1**–**8**) bearing ester groups of different geometries. Sal-**A**, Sal-**B**, and compounds **1**–**8** were tested against a human colorectal cancer cell line model with differing p53 status; HCT116 with wild-type p53 and HCT116 p53^−/−^ null for p53, and the normal-like human colon mucosa cells with wild-type p53, NCM460. IC_50_ values indicated that derivatization of Sal-**A** and Sal-**B** resulted in potentiation of HCT116 cell growth inhibition by 97% and 66%, respectively. The effects of the different molecules on cancer cell growth were independent of p53 status. Interestingly, the derivatization of Sal-**A** and Sal-**B** molecules enhanced their anti-growth properties versus 5-Fluorouracil (5-FU), which is the drug of choice in colorectal cancer. Structure-activity analysis revealed that the enhanced molecule potencies were mainly attributed to the position and number of the hydroxy groups, the lipophilicity, and the superiority of ester groups over hydroxy substituents in terms of their branching and chain lengths. The favorable cytotoxicity and selectivity of the potent molecules, to cancer cells versus their normal counterparts, pointed them out as promising leads for anti-cancer drug design.

## 1. Introduction

Terpenes have attracted considerable attention of medicinal chemists due to their numerous biological activities [1,2]. Sesquiterpene lactones (STLs) are a group of compounds commonly isolated from various genera of the Asteraceae family and are known to possess diverse biological activities [3]. In addition, several STLs have been tested as lead drugs in cancer clinical trials [4]. Therefore, the cytotoxicity and structure-activity relationship of STLs are the subject of intense interest. The biological activity of these complex plant secondary metabolites appears to be associated with their ability to act as alkylating agents due to a Michael addition reaction by biological nucleophiles to the α-methylene part of the lactone moiety [5,6]. α-methylene-γ-lactone was shown to react rapidly with cysteines to form stable adducts, whereas the endocyclic α, β-unsaturated-γ-lactones reacts slowly with cysteines to form unstable adducts [7]. It was also postulated that the reactions between α-methylene-γ-lactone and other conjugated systems with biologically crucial sulfhydryl groups may play a significant role in the mechanism by which these compounds exert their biological activities [7]. It was also observed that an increase in cytotoxicity accompanies an increase in lipophilicity. Furthermore, the presence of a bi-functionality, that is, either a cyclo-pentenone ring, α-methylene-δ-lactone, or a conjugated side chain ester in addition to α-methylene-γ-lactone increases cytotoxicity [8]. The importance of bi-functionality was also revealed in synthesized α-methylene-γ-butyrolactones [9]. Thus, STLs may exert their biological effects by inhibiting cellular enzyme activity and not by alkylating or impairing DNA function [10].

*Centaurea ainetensis (C. ainetensis)*—a Lebanese endemic plant that usually grows in stony, semi-arid regions—was reported previously by our group to possess anti-fungal [11], anti-proliferative, anti-tumor [12,13,14], and anti-inflammatory activities [15,16,17]. Following bioassay guided fractionation, we purified, identified, and characterized two STL molecules of the guaianolide group, salograviolide **A** (Sal-**A**) and salograviolide **B** (Sal-**B**), with promising anti-tumor and anti-inflammatory activities. Sal-**A** and Sal-**B** were also isolated from the aerial parts of another *Centaurea* species [18,19,20,21]. Sal-**A** possesses diverse biological activities such as anti-tumor and anti-inflammatory activities as previously reported [12,13,14,15]. However, the anti-cancer properties of Sal-**B** have not yet been investigated. In addition, many studies have compared the effects of different functional groups on either the cytotoxicity, specificity, or both, of STLs on a particular enzyme [22,23,24]. In this study, we evaluated the anti-growth activities of Sal-**B** and synthesized derivatives of Sal-**A** and Sal-**B** to develop more potent anti-cancer therapeutic leads compared to the parent molecules. Therefore, we prepared a new series of molecularly modified Sal-**A** and Sal-**B** derivatives by esterifying the OH-substituent(s). The potency of these compounds was compared to 5-Fluorouracil (5-FU), an antimetabolite fluoropyrimidine analog of the nucleoside pyrimidine with antineoplastic activity, the most commonly used anticancer drug in the treatment of colorectal cancer [25,26]. The anti-growth activities of the derivatives were tested against human colorectal cancer cell lines with wild-type and null p53 status to establish the relevant structural requirements to obtaining more potent and selective modified STLs. The tumor suppressor p53 is deregulated in at least 50% of colorectal cancers [27,28]. Colorectal cancer cells and tumors with no p53 activity are more aggressive and resistant to chemotherapy [27,29].

## 2. Discussion and Results

### 2.1. Synthesis of Salograviolide Derivatives

The aerial parts of *C. ainetensis* were air-dried at room temperature and then extracted with methanol. The organic extract was partitioned between water and chloroform. Repeated chromatographic purification of the chloroform extract yielded two known guanine sesquiterpenes, Sal-**A** and Sal-**B**. The structures of Sal-**A** [18,19,20,30] and Sal-**B** [18,19,20,21,31,32,33,34,35,36] were secured on the basis of an extensive comparison of their spectral data with those reported in the literature. To our knowledge, this is the first report of Sal-**B** purified from *C. ainetensis*, although it has been found in other Asteraceae plants [31,32,34,35,36].

The positive role of a hydroxy group close to the Michael acceptor had already been shown to enhance anti-inflammatory activity [37,38]. Hence, to evaluate the importance of the hydroxy group at positions C-8 and C-9 in Sal-**A** and at C-8 in Sal-**B**, a series of ester derivatives of Sal-**A** and Sal-**B** were examined for correlation between structural changes and biological activity. Modifiers such as acyl groups in the ester derivatives can rotate freely and thus would not affect the flexibility of the derivatives especially when approaching nucleophilic centers of biomolecules. In addition, they present new binding sites and control the hydrophobicity of the derivatives [39]. Increased lipophilicity should positively aid in the transportation of the derivatives through the cell membrane and might increase their anti-tumor properties but also enhance cytotoxicity [8]. Therefore, the search for the adequate chain length or the suitable molecular geometry to increase lipophilicity without significantly increasing cytotoxicity becomes important. In other words, the optimum Sal-**A** or Sal-**B** derivatives would exhibit a maximized anti-growth activity while maintaining their cytotoxicity at a minimum. Once absorbed into the cell, such acyl groups might be hydrolyzed in the cell by some esterases back to the original Sal-**A** or Sal-**B [40]**.

**Figure 1:**
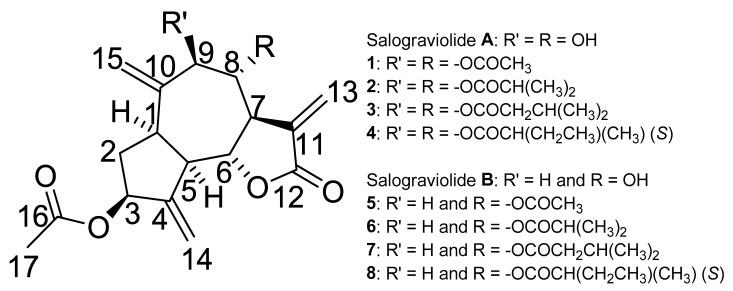
Structures of salograviolide **A**, **B** and their ester derivatives (**1**–**8**).

The ester series of acetate, isopropylate, isovalerate, and isobutyrate acyl groups of varying chain length and branching were used to esterify the hydroxy groups at positions C-8 and C-9 in Sal-**A** and at C-8 in Sal-**B**. Eight novel compounds (**1**–**4** from Sal-**A** and **5**–**8** from Sal-**B**) were synthesized (Figure 1, and supplementary material), purified, isolated, and tested. The standard method includes the treatment of Sal-**A** and Sal-**B** with the relative carboxylic acid anhydride (RCOOCOR), tri-ethylamine (Et_3_N), and 4-dimethylaminopyridine (DMAP) in chloroform produced the respective esters **1**–**4** and **5**–**8**. The structures of all synthesized salograviolide derivatives were confirmed by ^1^H-NMR, ^13^C-NMR, and MS spectra. The purity of all compounds was higher than 95%, verified by HPLC before evaluation of their biological efficacies (please see supplementary material for more details).

### 2.2. Anti-Growth Effects of Sal-**A**, Sal-**B**, and Derivatives against Colorectal Cancer In Vitro Model and Structure-Activity Analysis

**Figure 2:**
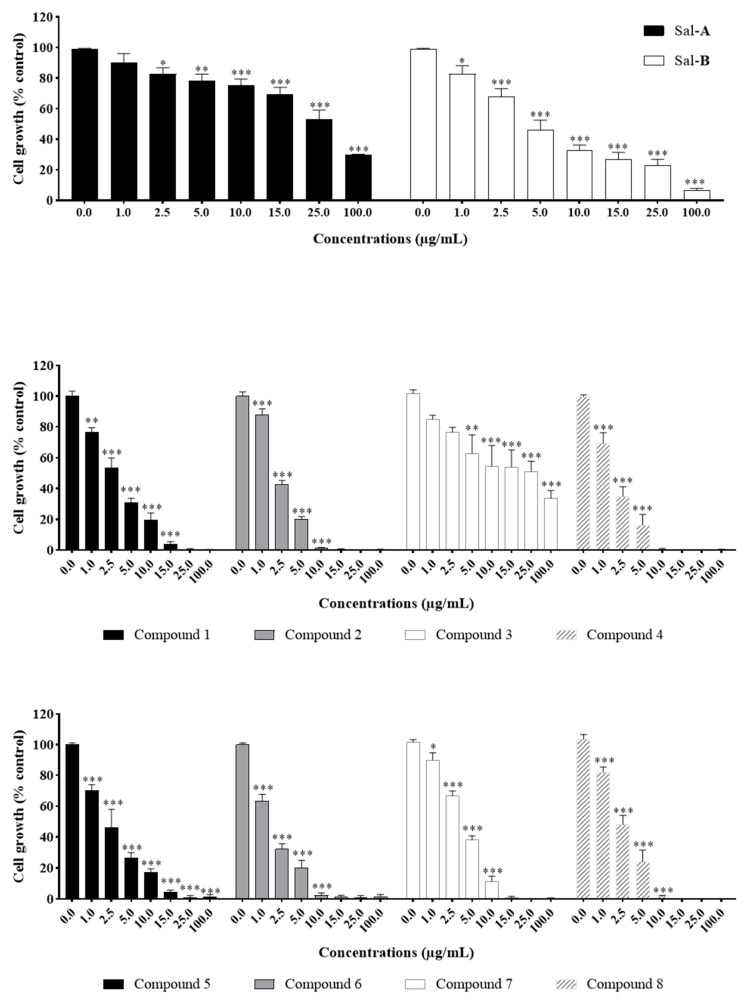
Effect of Sal-**A**, Sal-**B**, and their derivatives on the growth of HCT116 colorectal cancer cells. Cells were treated with 1% DMSO (control) or the indicated concentrations of the compounds for up to 48 h. Cell growth was determined in triplicate wells using the MTT cell proliferation assay. Results are expressed as percentage of control and represent the average of three independent experiments ± SEM. Statistical significance is reported by two-way ANOVA post hoc Bonferroni’s multiple comparisons test indicating differences between treated cells and control at 48 h post-treatment (*, *p* < 0.05; **, *p* < 0.01; ***, *p* < 0.001).

**Figure 3:**
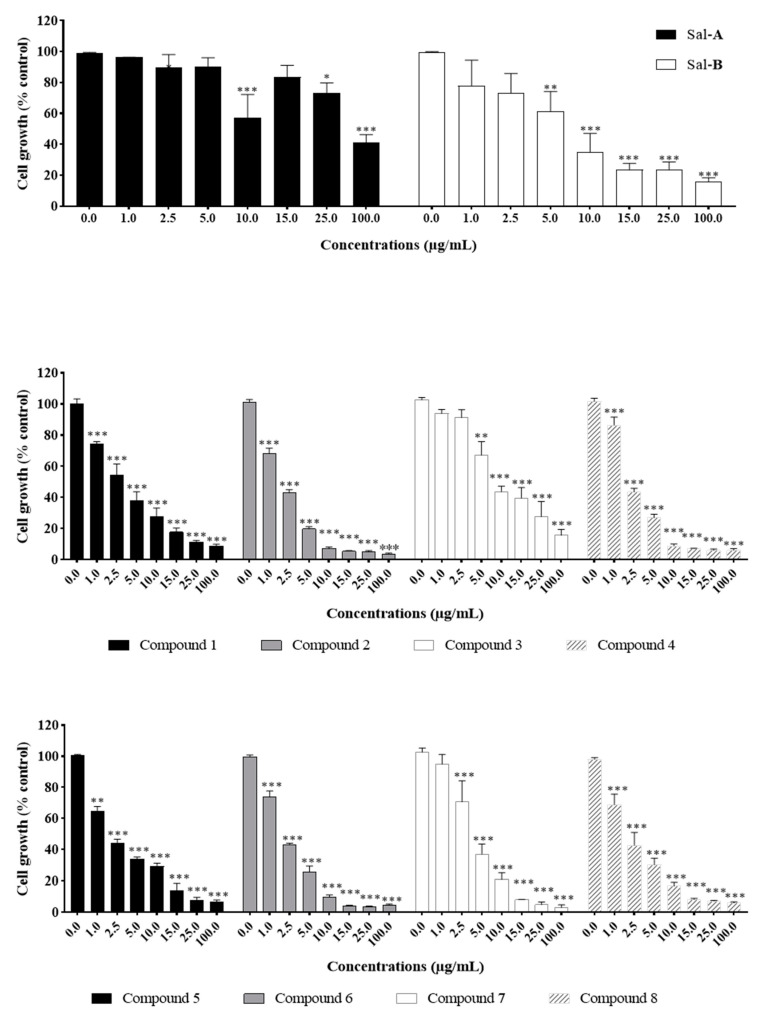
Effect of Sal-**A**, Sal-**B**, and their derivatives on the growth of HCT116 p53^−/−^ colorectal cancer cells. Cells were treated with 1% DMSO (control) or the indicated concentrations of the compounds for up to 48 h. Cell growth was determined in triplicate wells using the MTT cell proliferation assay. Results are expressed as percentage of control and represent the average of three independent experiments ± SEM. Statistical significance is reported by two-way ANOVA post hoc Bonferroni’s multiple comparisons test indicating differences between treated cells and control at 48 h post-treatment (*, *p* < 0.05; **, *p* < 0.01; ***, *p* < 0.001).

**Figure 4:**
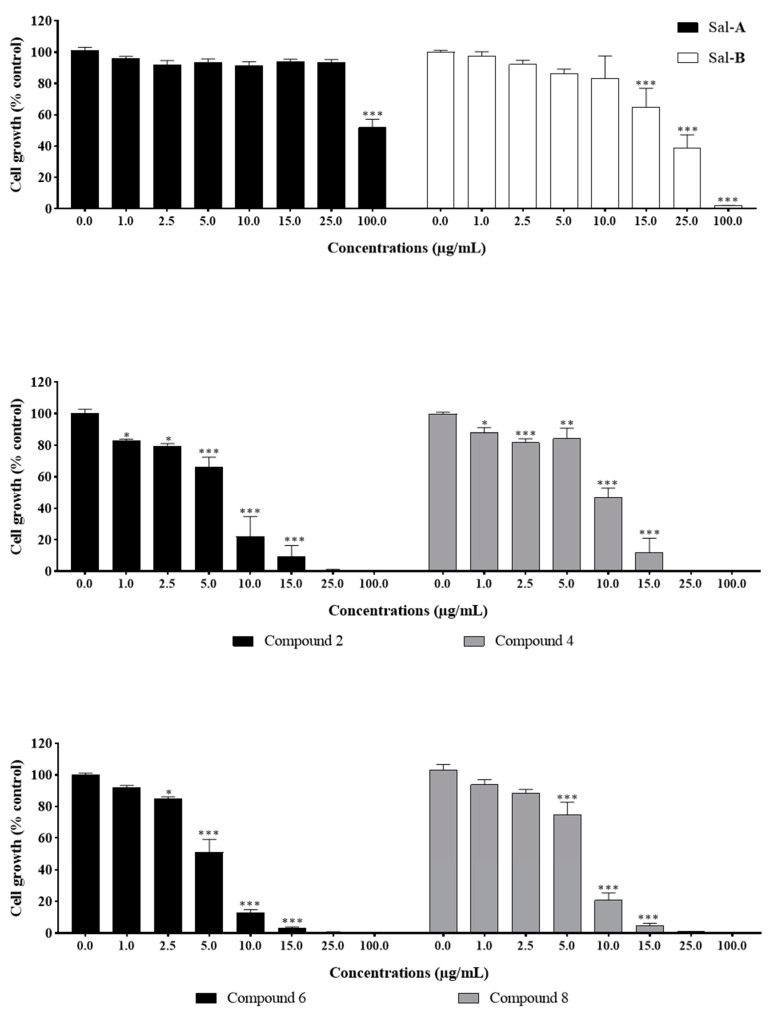
Effect of Sal-**A**, Sal-**B**, and their most potent derivatives on the growth of the normal-like NCM460 cells. Cells were treated with 1% DMSO (control) or the indicated concentrations of the compounds for up to two days. Cell growth was determined in triplicate wells using the MTT cell proliferation assay. Results are expressed as percentage of control and represent the average of three independent experiments ± SEM. Statistical significance is reported by two-way ANOVA post hoc Bonferroni’s multiple comparisons test indicating differences between treated cells and control at 48 h post-treatment (*, *p* < 0.05; **, *p* < 0.01; ***, *p* < 0.001).

**Figure 5:**
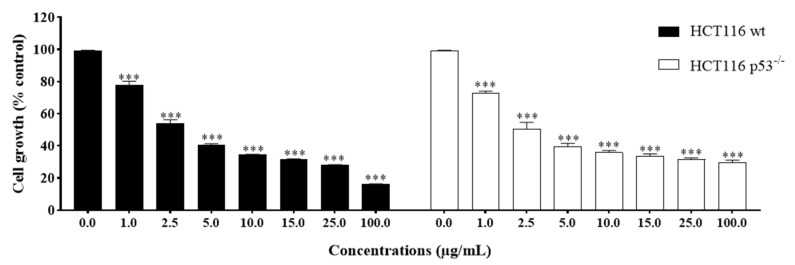
Effect of 5-Fluorouracil on the growth of HCT116 and HCT116 p53^−/−^cells. Cells were treated with 0.1% DMSO (control) or the indicated concentrations of the compounds for up to two days. Cell growth was determined in triplicate wells using the MTT cell proliferation assay. Results are expressed as percentage of control and represent the average of three independent experiments ± SEM. Statistical significance is reported by two-way ANOVA post hoc Bonferroni’s multiple comparisons test indicating differences between treated cells and control at 48 h post-treatment (***, *p* < 0.001).

The anti-growth effects of Sal-**A**, Sal-**B**, and the eight synthesized derivatives (**1**–**8**) were evaluated in an in vitro model of human colorectal cancer using the 3-(4,5-dimethylthiazol-2-yl-),5-diphenyl tetrazolium bromide (MTT) dye-reduction assay as previously described [41]. This cell line model consists of the HCT116 wild type for p53 and HCT116 p53^−/−^ null for p53 cells and the normal-like human colon mucosa cells, NCM460, with wild-type p53. The concentrations that induce 50% cell growth inhibition (IC_50_) are reported in Table 1. The tested derivatives displayed more potent anti-growth effects compared with the parent molecules (Sal-**A** and Sal-**B**). Compounds **2**, **4**, **6**, and **8** were found to be the most potent, while **3** and **7** were the least effective among the derivatives but more than the parent molecules (Figure 2 and Figure 3). These active derivatives showed minor anti-growth activity against the normal-like NCM460 cells, indicating differential properties in cancer versus normal cells (Figure 4). The potency of these compounds was then compared to 5-FU on both HCT116 and HCT116 p53^−/−^ cells (Figure 5). Remarkably, all the tested compounds, except the parental compound Sal-**A,** displayed a higher anticancer effect at lower concentrations then 5-FU (approximate IC_50_ = 19.2 µM at 48 h of treatment). The properties and biological potencies against HCT116 and HCT116 p53^−/−^ of Sal-**A**, Sal-**B**, and their derivatives were analyzed based on the bearing molecular functional groups and their chain geometry.

### 2.3. The Potency of Sal-**A**, Sal-**B**, and Their Derivatives on Colorectal Cancer Cells Is Not Due to Cell Toxicity

**Figure 6:**
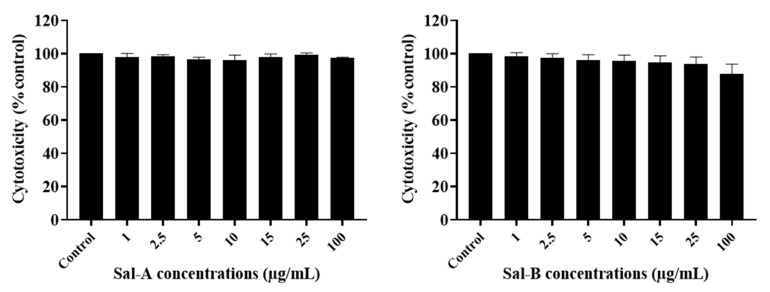
Cytotoxic effect of Sal-**A** and Sal-**B** on HCT116 cells. Cells were treated with 1% DMSO (control) or the indicated concentrations of the compounds for 6 h. The cytotoxic activity was determined in triplicate wells by the lactate dehydrogenase assay. Results are expressed as percentage of control and represent the average of three independent experiments ± SEM.

**Figure 7:**
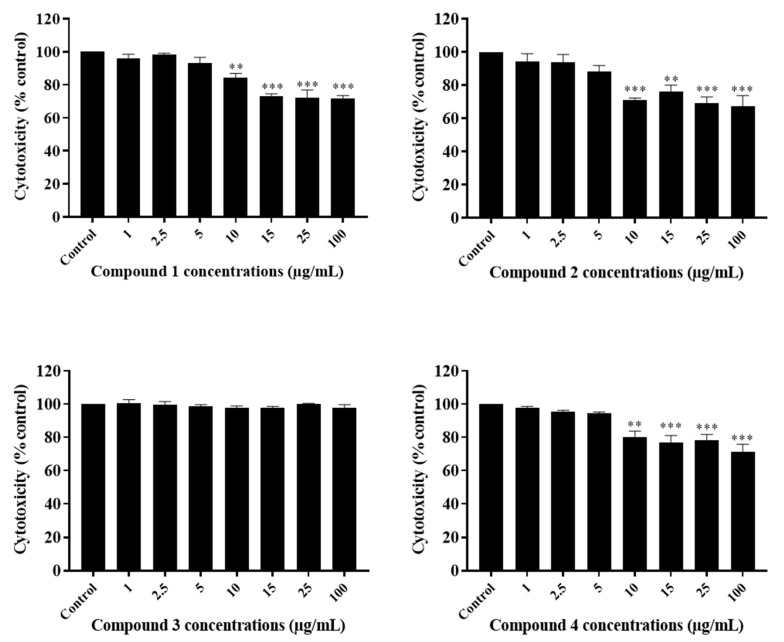
Cytotoxic effect of Sal-**A** derivatives on HCT116 cells. Cells were treated with 1% DMSO (control) or the indicated concentrations of the compounds for six hours. The cytotoxic activity was determined in triplicate wells by the lactate dehydrogenase assay. Results are expressed as percentage of control and represent the average of three independent experiments ± SEM. Statistical significance is reported by one-way ANOVA post hoc Tukey test indicating differences between treatment concentrations and control (**, *p* < 0.01; ***, *p* < 0.001).

**Figure 8:**
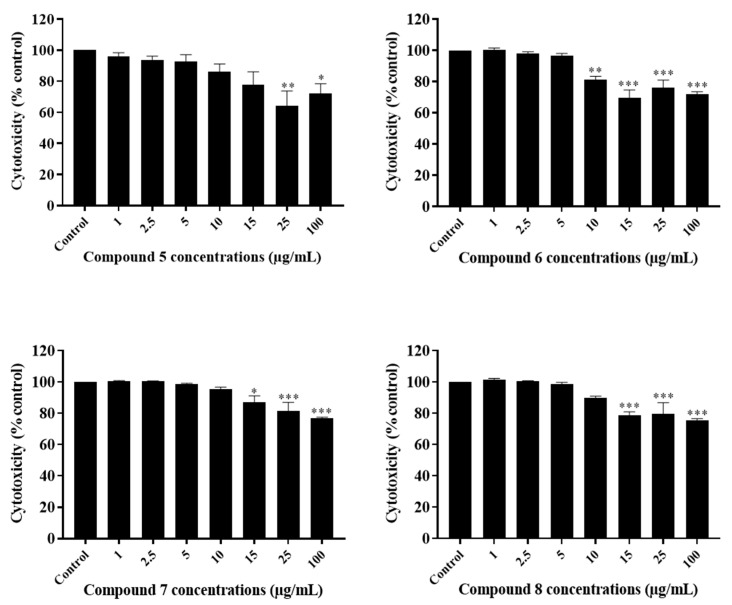
Cytotoxic effect of Sal-**B** derivatives on HCT116 cells. Cells were treated with 1% DMSO (control) or the indicated concentrations of the compounds for six hours. The cytotoxic activity was determined in triplicate wells by the lactate dehydrogenase assay. Results are expressed as percentage of control and represent the average of three independent experiments ± SEM. Statistical significance is reported by one-way ANOVA post hoc Tukey test indicating differences between treatment concentrations and control (*, *p* < 0.05; **, *p* < 0.01; ***, *p* < 0.001).

The cytotoxicity of the tested compounds on HCT116 cells was quantitatively measured at six hours post-treatment. It was assessed by the measurement of the activity of the stable cytosolic lactate dehydrogenase (LDH) enzyme released to the outer milieu after lysis of damaged cells. When quantified at early time points, this assay allows the detection of the acute effect of the drug as highlighted by cell bursting and LDH release. Cells were treated with the previously selected concentrations. Sal-**A**, Sal-**B**, and compound **3** were non-cytotoxic up to 100 µg/mL, while a significant cytotoxic effect was noted starting at concentrations of 10–15 µg/mL for the other derivatives (Figure 6, Figure 7 and Figure 8). However, these concentrations are at least two folds higher than the previously calculated IC_50_ values.

### 2.4. Comparing Potencies between Sal-**A** and Sal-**B**: Effect of Hydroxy (OH)-Group at the C-8 Position on Colorectal Cancer Cell Growth

The presence of an OH group at the C-8 position next to the α-methylene-γ-lactone moiety in both Sal-**A** and Sal-**B** influenced the potency of both molecules as previously reported [8,42,43,44]. Neighboring OH groups to the molecule’s main alkylating center have been shown to enhance the rate of cysteine addition by presumably facilitating the incorporation of the sulfur anion RS− or the proton transfer at an intermediate stage in the Michael-type addition reaction [8]. Sal-**B** showed enhanced potency over Sal-**A** (IC_50_ of 4.4 vs. 31.9 for HCT116, and 5.1 versus 69.5 for HCT116 p53^−/−^) a property attributed to a lower number of OH groups. An increasing number of free OH groups, as in the case of Sal-**A,** seems to decrease the growth inhibitory activity. A lower number of OH groups correlates with higher lipophilicity followed by enhanced penetration through the cell membranes. Sal-**B** is more hydrophobic than Sal-**A** as predicted by the calculated log P values (log P of 10.35 mg/mL and 1.06 mg/mL for Sal-**A** and Sal-**B**, respectively). This is in agreement with previous reports suggesting the existence of an optimal number of OH groups for STL activity [43].

### 2.5. Neighboring O-Acyl Group Affects Cysteine Addition Rate

The substitution of OH groups by ester functions positioned at C-8 and C-9 in Sal-**A** and at C-8 in Sal-**B** showed an increase in the potencies of compounds **1**–**4** in comparison with Sal-**A** and compounds **5**–**8** when contrasted to Sal-**B**. Relative to Sal-**A**, the IC_50_ of compounds **1**, **2**, **3**, and **4** decreased by 90%, 92%, 78%, and 94%, respectively. Derivatives of Sal-**B** were also more potent with IC_50_ decreasing by 50% for compound **5**, 59% for **6**, 14% for **7**, and 50% for **8.** These findings are in agreement with Kupchan et al. [8] who found that the neighboring O-acyl group produced a marked enhancement in the rate of cysteine addition. Therefore, the existence of such number of ester groups in one molecule seems to be an important feature for the growth inhibitory activity.

### 2.6. Steric Hindrance Effects: Lipophilicity and Tertiary Alpha Carbon

The carbon branching of the alkyl chain in the different types of ester derivative seems to have a unique effect on the anti-growth properties. An increase, by one carbon, in the carbon branch from compounds **1** to **2** and **5** to **6** correlates with enhanced potency of the sought derivatives by up to 30%. However, when an additional aliphatic carbon was added to the branch, as it is the case in **3** and **7**, potencies lower by approximately 60% than the ones calculated for compounds **2** and **6** were observed. This behavior is attributed to a steric hindrance caused by the isovaleryl group. The bulkiness of the molecule is mostly shown in the least active compound **7** where two isovaleryl groups at C-8 and C-9 are present. In line with previous literature findings, the lipophilic enhancement obtained using larger ester groups is size-limited by steric hindrance effects on the exocyclic methylene group, preventing it from approaching its target [45]. For example, in the bifunctional helenalin and mexicanin I analogs, the addition of a lipophilic character enhanced cytotoxicity against Ehrlich ascites carcinoma in vitro and in vivo; however, there was a size optimum of lipophilic ester groups beyond which STL toxicity decreased [44,45].

Interestingly, the pronounced anti-growth effect of the isopropyl and isobutyl esters (**2**, **4**, **6**, and **8**) to HCT116 and HCT116 p53^−/−^ cell lines might be attributed to the presence of a tertiary alpha-carbon in the ester group. The same structure seems to also enhance the cytotoxicity by up to 75% than the primary (**1** and **5**) and secondary (**3** and **7**) alpha-carbon.

## 3. Conclusions

In the current work, a series of eight new guaianolide-type STLs derivatives were synthesized with ester group(s) modification at either C8-OH, C9-OH, or both, positions. All of the derivatives examined for their inhibition of colorectal cancer cell growth showed that compounds with isopropyl and isobutyl chains were active against HCT116 and HCT116 p53^−/−^ cells. The difference in anti-growth potency between the synthesized derivatives (compounds **1**–**8**) and their parent compounds (Sal-**A** and -**B)** suggests a positive contribution of the ester chain at C-8 and C-9 and confirms the ability of STLs to inhibit colorectal cancer cell growth independently of p53 status. Unspecific cell lysis, as demonstrated by LDH release into the medium, is observed at concentrations about 3–4-times the IC_50_ against the normal control cells and at concentrations about 3–6 times against the cancer cells. This finding indicates that the new guaianolide-type STLs derivatives have a specific cell death effect on colorectal cancer cells. Colorectal cancer is one of the most common and deadliest cancers worldwide and there is an urgent need for novel therapies [46,47] It would be interesting to test the effect of the most active derivatives on colorectal cancer cell lines with mutant p53 as the majority of observed mutations in human cancers are missense-type, some of them conferring oncogenic “gain-of-function” mechanisms [28].

Although the target enzymes/proteins affected by the STLs in the tested cell lines used in this study are not yet known, our findings demonstrate that the position of the hydroxy group at C-8, the lipophilicity, the nature of substituent group (OH versus ester), the carbon chain size, and the presence of tertiary alpha-carbon in the ester groups, are all relevant factors to be considered in the interpretation of STL biological activity. These findings highlight the fact that potent ester derivatives adjacent to the α-methylene-δ-lactone in STLs at the C-8 and C-9 positions need to be lipophilic, of an optimal size, and have a tertiary alpha-carbon in the ester group. Interestingly, the derivatization of Sal-**A** and Sal-**B** molecules enhanced their anti-growth properties versus 5-FU which is the drug of choice in colorectal cancer therapy for the last six decades.

Taken together, the introduction of ester groups into Sal-**A** and Sal-**B** enhanced the growth suppressive activity and improved the selectivity compared with the corresponding parent compounds and increased their potency versus 5-FU. Importantly, this study provides insight into developing novel anti-cancer compounds with high degree of selectivity for cancer versus normal cells. Based on these structure-activity relationships, further optimization—in terms of engineering other ester-based derivatives with tertiary α-carbons and even quaternary ones—and evaluating their biological activity and cell death mechanism of action as anti-colorectal cancer growth inhibitors are ongoing in our laboratory.

## 4. Materials and Methods

### 4.1. General Experimental Procedures

Ultraviolet-visible (UV-Vis) spectra were measured in methanol using a Jasco V-570 UV/VIS/NIR spectrophotometer. Infrared red (IR) spectra were recorded using a Nicolet AVATAR 360 Fourier transform infrared (FTIR) spectrometer equipped with a KBr pellet cell holder. ^1^H and ^13^C (500 MHz) nuclear magnetic resonance (NMR) spectra were measured on a Bruker Avance III HD 500 NMR spectrometer. High-performance liquid chromatography (HPLC) was performed on an automatic thermostated column compartment housing a C18 reversed-phase column (250 × 4.6 mm i.d.; 5 μm, Supelco). Preparative HPLC was performed on a Gilson GX-271 Prep (150 × 50 mm i.d.; 10 μm) Reprosil 100 C18 column. The pre-column is a Reprosil 100 C18 column (50 × 50 mm i.d.; 10 μm) coupled with ultraviolet detection (UV/VIS-156). All chemicals were purchased from Sigma-Aldrich Co. (St. Louis, MO, USA). For extraction, we used methanol (MeOH) (ACS reagent, Sigma-Aldrich Chemie GmbH, St. Quentin Fallavier Cedex, France), n-hexane (analytical grade, VWR Prolabo Chemicals, France), chloroform (CHCl_3_) (Sigma-Aldrich Chemie GmbH, Taufkirchen, Germany), and ethylacetate (EtoAc) (Sigma-Aldrich Chemie GmbH, Taufkirchen, Germany). For purification, HPLC grade water (CHROMASOLV^@^ gradient grade, Sigma-Aldrich Chemie GmbH, Schaffhausen, Switzerland), HPLC grade acetonitrile (CH_3_CN) (CHROMASOLV^@^ gradient grade, Sigma-Aldrich Chemie GmbH, Taufkirchen, Germany) were used for analytical RP-HPLC and preparative HPLC. Deuterated solvents for NMR were purchased from Cambridge isotope laboratories, Inc. (Andover, MA, USA).

### 4.2. Plant Material

The plant material of *C. ainetensis* was collected from the Bcharre Cedars area in Northern Lebanon at an altitude of 1650 m during the flowering stage in June 2018 (GPS 34°11′56.74″ N, 36°5′14.60″ E). Voucher specimens were deposited in the herbarium of the Faculty of Agriculture and Food Sciences at the American University of Beirut (Beirut, Lebanon). The aerial parts were dried by leaving the plant samples in the shade for two weeks before grinding into approximately 10 mm pieces using a blender.

### 4.3. Extraction and Isolation

Extracts from the aerial parts of *C. ainetensis* (300 g) were prepared as previously described [16]. Briefly, the aerial parts were soaked, separately, in 2 L methanol for 16 h at room temperature. The crude methanolic extracts (given the name “I”) were concentrated to 1/10 of their volumes and acidified to pH = 2 with a sulfuric acid solution. Liquid-liquid extraction using a mixture of CHCl_3_: water (2:1 *v*/*v*) followed and the organic layer (called “I.2”) was collected and evaporated under reduced pressure at 40 °C to give 5.0 g. The fraction I.2 was assayed for its anti-growth and cytotoxic activity. It was also applied to a liquid column chromatography (silica gel 0.035–0.075 mm, 60 Å, 500 g) and fractionated using a gradient elution of petroleum ether: CHCl_3_:EtOAc (2:1:2), followed by petroleum ether: CHCl_3_: EtOAc (2:2:1), CHCl_3_: EtOAc:MeOH(3:3:1), CHCl_3_: MeOH (3:2), and MeOH. The 14 fractions obtained from (“I.2.1–I.2.14”), were further purified by preparative HPLC. Fraction I.2.10, which was separated by preparative HPLC, (50:50 ACN: H_2_O, flow rate of 10 mL/min with detection at 214 nm) yielded pure Sal-**A** (1 g). However, fraction I.2.11, (preparative HPLC, 50:50 ACN: H_2_O, flow rate of 10 mL/min with detection at 210 nm) gave pure Sal-**B** (100 mg). This procedure was repeated multiple times in order to isolate the needed amounts of for Sal-**A** and Sal-**B** for further derivatization.

### 4.4. Derivatization

Triethylamine (Et_3_N) (0.5 mmol) was added to a mixture of Sal-**A** and Sal-**B** (0.1 mmol each) and DMAP (5 mg) in dichloromethane (CH_2_Cl_2_) (5 mL). The reaction mixture was cooled to 0 °C and treated with acetic anhydride (0.5 mmol) to prepare compounds **1** and **5**, with isobutyric anhydride (0.5 mmol) to prepare compounds **2** and **6**, with isovaleric anhydride (0.5 mmol) to prepare compounds **3** and **7**, and with (*S*)-(+)-2-methylbutyric anhydride (0.5 mmol) to prepare compounds **4** and **8**. The resulting solution was warmed to room temperature for 12 h. After completion, the reaction mixture was diluted with CH_2_Cl_2_ (10 mL), washed with water, and brine. The organic layer was dried over Na_2_SO_4_, filtered, and concentrated in vacuo. The residues were purified via preparative HPLC (70:30 ACN: H_2_O, flow rate of 10 mL/min with detection at 210 nm and 214 nm) to give quantitative yields of the different derivatives.

Salograviolide **A.** Colorless needless (CHCl_3_); UV (MeOH) λ_max_ (log ε): 214 (2.70), 220 (3.00) nm; IR (KBr) ν_max_: 3430, 1762, 1730, 1660, 1616 cm^−1^; ^1^H-NMR (CDCl_3_, 500 MHz) δ 6.40 (1H, dd, *J* = 1.17, 3.15 Hz, H-13a), 6.35 (1H, dd, *J* = 1.17 Hz, H-13b), 5.55 (1H, m, H-3), 5.50 (1H, d, *J* = 0.88 Hz, H-14a), 5.20 (1H, *J* = 0.88 Hz, H-14b), 5.47 (1H, t, *J* = 1.78 Hz, H-15a), 5.31 (1H, t, *J* = 3.78 Hz, H-15b), 3.97 (1H, dd, *J* = 8.06 Hz, CHOH, H-9), 3.93 (1H, dd, *J* = 9.48, 9.37 Hz, H-6), 3.59 (1H, m, OH), 3.46 (1H, ddd, *J* = 10.38, 8.06 Hz, CHOH, H-8), 3.08 (1H, m, OH), 2.97 (1H, ddd, *J* = 9.37, 3.15, 10.38 Hz, H-7), 2.92 (1H, m, H-1, H-5), 2.58 (1H, m, H-2a), 2.13 (3H, s, OCH_3_-1’), 1.85 (1H, ddd, H-2b); ^13^C-NMR (CDCl_3_, 500 MHz) δ 170.7 (C, C-16), 169.8 (C, C-12), 147.8 (C, C-11), 147.4 (C, C-4), 135.9 (C, C-10), 125.6 (CH_2_, C-13), 113.3 (CH_2_, C-14), 112.8 (CH_2_, C-15), 79.9 (CH, C-9), 79.3 (CH, C-6), 77.7 (CHOH, C-8), 74.5 (CH, C3), 48.9 (CH, C-1), 47.2 (CH, C-5), 41.1 (CH, C-7), 36.3 (CH_2_, C-2), 21.3 (CH_3_, OCOCH_3_-17); (+) EIMS *m*/*z* 320 (M)^+^; HREIMS *m*/*z* 320.1260, calculated for C_17_H_20_O_6_ *m*/*z* 320.1260.

Salograviolide **B.** Colorless needless (CHCl_3_); UV (MeOH) λ_max_ (log ε): 210 (2.90), 213 (3.50) nm; IR (KBr) ν_max_: 3503, 1760, 1731, 1659 cm^−1^; ^1^H-NMR (CDCl_3_, 500 MHz) δ 6.28 (1H, d, *J* = 3.4 Hz, H-13a), 6.16 (1H, d, *J* = 3 Hz, H-13b), 5.55 (1H, m, *J* = 7.5, 7.5, 2.2, H-3), 5.52 (1H, br s, H-15a), 5.33 (1H, br s, H-15b), 5.12 (1H, br s, H-14a), 5.00 (1H, br s, H-14b), 4.10 (1H, dd, *J* = 10.5, 9 Hz, H-6), 4.00 (1H, br m, OH), 3.99 (1H, ddd, *J* = 9,5,4 Hz, H-8), 3.03 (1H, ddd, *J* = 11, 8, 8 Hz, H-1), 2.82 (1H, br dd, *J* = 9.8 Hz, H-5), 2.79 (1H, m, *J* = 10.5, 9, 3.4, 3 Hz, H-7), 2.67 (1H, dd, *J* = 14, 5 Hz H-9b), 2.36 (1H, ddd, *J* = 13.5, 7.5, 7.5 Hz, H-2b), 2.29 (1H, dd, *J* = 14, 4 Hz H-9a), 2.09 (3H, s, OCH3-1’), 1.78 (2H, ddd, *J* = 13.5, 11, 7.5 Hz, H-2a); ^13^C-NMR (CDCl_3_, 500 MHz) δ 170.8 (C, C-16), 169.6 (C, C-12), 147.3 (C, C-4), 142.2 (C, C-10), 137.9 (C, C-11), 123.3 (CH_2_, C-13), 117.5 (CH_2_, C-15), 115.8 (CH_2_, C-14), 78.1 (CH, C-6), 74.6 (CH, C-3), 71.9 (CHOH, C-8), 51.7 (CH, C-5), 51 (CH, C-7), 45.6 (CH, C-1), 41.4 (CH_2_, C-2), 36.3 (CH_2_, C-9), 21.2 (CH_3_, OCOCH_3_-17); (+) EIMS *m*/*z* 305 (M)^+^; HREIMS *m*/*z* 305.11699, calculated for C_17_H_21_O_5_ *m*/*z* 305 *m*/*z* 305.1161.

Compound **1**. Yield: 30 mg (74%). Colorless needless (CHCl_3_); UV (MeOH) λmax (log ε)_:_ 214 (2.70) nm. ^1^H-NMR (CDCl_3_, 500 MHz) δ 6.30 (1H, d, *J* = 3.30 Hz, H-13a), 5.64 (1H, d, *J* = 2.95 Hz, H-13b), 5.58 (1H, m, H-3), 5.47 (1H, t, *J*_1_ = 2.20 Hz, *J*_2_ = 2.20 Hz, H-14a), 5.38 (1H, s, H-9), 5.32 (1H, t, *J*_1_ = 2.15 Hz, *J*_2_ = 2.15 Hz, H-14b), 5.22 (1H, s, H-8), 5.17 (2H, m, H-15a, 15b), 4.09 (1H, t, *J* = 9.15, 0.55, 9.20 Hz, H-6), 3.19 (1H, m, H-7), 3.05 (1H, m, H-5), 2.95 (1H, m, H-1), 2.57 (1H, ddd, *J* = 8.60, 2.3, 8.60 Hz, H-2a), 2.13 (3H, s, OCOCH3-17), 2.10 (3H, s, OCOCH_3_-2’), 2.07 (3H, s, OCOCH_3_-4’), 1.85 (1H, m, H-2b). ^13^C-NMR (CDCl_3_, 500 MHz): δ 170.57 (C, C-16), 169.60 (C, C-1’), 169.39 (C, C-3’), 168.77 (C, C-12), 147.24 (C, C-10), 142.82 (C, C-4), 135.01 (C, C-11), 124.25 (CH_2_, C-13), 114.97 (CH_2_, C-14), 113.88 (CH_2_, C-15), 79.14 (CH, C-9), 77.95 (CH, C-6), 74.70 (CH, C-3), 74.39 (CH, C-8), 49.19 (CH, C-5), 45.27 (CH, C-7), 41.33 (CH, C-1), 36.38 (CH_2_, C-2), 21.22 (CH_3_, OCOCH_3_-17), 20.93 (CH_3_, OCOCH_3_-2’), 20.87 (CH_3_, OCOCH_3_-4’); (+) EIMS (*m*/*z*) 427.0 (MNa^+^), calculated for C_21_H_24_NaO_8_, 427.1.

Compound **2.** Yield: 40 mg (88%). Colorless needless (CHCl_3_); UV (MeOH) λmax (log ε)_:_ 214 (2.70) nm. ^1^H-NMR (CDCl_3_, 500 MHz) δ 6.28 (1H, d, *J* = 3.35 Hz, H-13a), 5.61 (1H, d, *J* = 3.13 Hz, H-13b), 5.59 (1H, m, H-3), 5.49 (1H, t, *J*_1_ = 2.20 Hz, *J*_2_ = 2.20 Hz, H-14a), 5.36 (1H, d, *J* = 1.0 Hz, H-15a), 5.34 (1H, t, *J*_1_ = 2.15 Hz, *J*_2_ = 2.15 Hz, H-14b), 5.25 (1H, t, *J* = 8.75, 1.75, 8.85 Hz, H-9), 5.21 (1H, s, H-8), 5.18 (1H, d, *J* = 8.80 Hz, H-15b), 4.09 (1H, t, *J*_1_ = 9.75 Hz, *J*_2 =_ 9.30 Hz, H-6), 3.22 (1H, m, H-7), 3.09 (1H, m, H-5), 2.97 (1H, m), 2.60 (2H, m, OCOCH-2’, 4’), 2.53 (1H, m, H-2a), 2.12 (3H, s, OCH_3_-17), 1.87 (1H, m, H-2b), 1.21 (3H, s, OCOCHCH_3_-5’), 1.20 (3H, s, OCOCHCH_3_-6’), 1.17 (3H, s, OCOCHCH_3_-7’), ^13^C-NMR (CDCl_3_, 500 MHz): δ 175.50 (C, C-1’), 175.38 (C, C-3’), 170.56 (C, C-16), 168.81 (C, C-12), 147.17 (C, C-10), 143.40 (C, C-4), 135.28 (C, C-11), 123.92 (CH_2_, C-13), 114.45 (CH_2_, C-14), 114.07 (CH_2_, C-15), 79.03 (CH, C-9), 77.66 (CH, C-6), 74.46 (CH, C-3), 74.09 (CH, C-8), 49.03 (CH, C-5), 45.90 (CH, C-7), 41.29 (CH, C-1), 36.26 (CH_2_, C-2), 34.16 (CH, OCOCH-4’), 33.96 (CH, OCOCH-2’), 21.21 (CH_3_, OCOCH_3_-17), 19.05 (CH_3,_ OCOCHCH_3_-5’), 18.87 (CH_3,_ OCOCHCH_3_-6’), 18.65 (CH_3,_ OCOCHCH_3_-7’), 18.57 (OCOCHCH_3_, C-8’); (+) EIMS (*m*/*z*) 483.2 (MNa^+^), calculated for C_25_H_32_NaO_8_, 483.2.

Compound **3.** Yield: 30 mg (61%). Colorless needless (CHCl_3_); UV (MeOH) λmax (log ε)_:_ 214 (2.70) nm. ^1^H-NMR (CDCl_3,_ 500 MHz) δ 6.29 (1H, d, *J* = 3.35 Hz, H-13a), 5.64 (1H, d, *J* = 2.90 Hz, H-13b), 5.59 (1H, m, H-3), 5.49 (1H, t, *J*_1_ = 2.20 Hz, *J*_2_ = 2.2 Hz, H-14a), 5.36 (1H, s, H-9), 5.33 (1H, t, *J*_1_ = 2.15 Hz, *J*_2_ = 2.15 Hz, H-14b), 5.22 (1H, d, *J* = 9.15 Hz, H-15a), 5.20 (1H, d, *J* = 8.85 Hz, H-8), 5.16 (1H, d, *J* = 9.00 Hz, H-15b), 4.09 (1H, t, *J* = 9.10, 0.8, 9.10 Hz, H-6), 3.19 (1H, m, H-7), 3.08 (1H, m, H-5), 2.96 (1H, m, H-1), 2.58 (1H, ddd, *J* = 8.55, 2.25, 8.55 Hz, H-2a), 2.25 (2H, d, *J* = 7.00 Hz, OCOCH_2_-2’), 2.19 (2H, t, *J*_1_ = 7.30 Hz, *J*_2_ = 6.75 Hz, OCOCH_2_-4’), 2.14 (1H, m, H-5’), 2.11 (3H, s, OCOCH_3_-17), 2.08 (1H, m, H-8’), 1.86 (1H, m, H-2b), 0.93-0.99 (12H, dd, *J* = 6.6 Hz, OCO CH_2_CHCH_3_-6’, 7’, 9’, 10’). ^13^C NMR (CDCl_3_, 500 MHz) δ 171.62 (C, C-1’), 171.56 (C, C-3’), 170.58 (C, C-17), 168.81 (C, C-12), 147.17 (C, C-10), 143.39 (C, C-4), 135.16 (C, C-11), 124.12 (CH_2_, C-13), 114.77 (CH_2_, C-14), 114.07 (CH_2_, C-15), 79.10 (CH, C-9), 77.76 (CH, C-6), 74.46 (CH, C-3), 74.08 (CH, C-8), 49.13 (CH, C-5), 45.75 (CH, C-7), 43.21 (CH, OCOCH_2_CH-5’), 41.37 (CH, OCOCH_2_CH -8’), 40.57 (CH, C-1), 36.32 (CH_2_, C-2), 29.70 (CH_2_, OCOCH_2_-2’), 25.53 (CH_2_, OCOCH_2_-4’), 25.52 (CH_3_, OCOCH_3_-17), 22.49 (CH_3_, OCOCH_2_CHCH_3_-6’), 22.45 (CH_3_, OCOCH_2_CHCH_3_-7’), 22.37 (CH_3_, OCOCH_2_CHCH_3_-9’), 21.22 (CH_3_, OCOCH_2_CHCH_3_-10’); (+) EIMS (*m*/*z*) 511.3 (MNa^+^), calculated for C_27_H_36_NaO_8_, 511.2.

Compound **4.** Yield: 30 mg (61%). Colorless needless (CHCl_3_); UV (MeOH) λmax (log ε)_:_ 214 (2.70) nm. ^1^H-NMR (CDCl_3_, 500 MHz) δ 6.28 (1H, d, *J* = 3.3 Hz, H-13a), 5.64 (1H, d, *J* = 2.9 Hz, H-13b), 5.58 (1H, m, H-3), 5.49 (1H, t, *J*_1_ = 2.15 Hz, *J*_2_ = 2.15 Hz, H-14a), 5.36 (1H, s, H-9), 5.34 (1H, t, *J*_1_ = 2.15 Hz, *J*_2_ = 2.15 Hz, H-14b), 5.25 (1H, ddd, *J* = 8.70, 1.75, 8.75 Hz, H-15a), 5.20 (1H, d, *J* = 0.55 Hz, H-8), 5.17 (1H, d, *J* = 8.75 Hz, H-15b), 4.09 (1H, ddd, *J* = 9.30, 0.55, 9.35 Hz, H-6), 3.20 (1H, m, H-7), 3.10 (1H, ddd, *J* = 5.50, 5.25, 5.45 Hz, H-5), 2.97 (1H, m, H-1), 2.56 (1H, ddd, *J* = 8.45, 2.15, 8.45 Hz, H-2a), 2.38 (2H, m, OCOCHCH_2_-5’), 2.11 (3H, s, OCOCH_3_-17), 1.86 (1H, ddd, *J* = 5.30, 4.20, 5.30 Hz, H-2b), 1.76 (1H, m, OCOCH-2’), 1.67 (1H, m, OCOCH-4’), 1.46 (2H, m, OCOCHCH_2_-8’), 1.18 (3H, d, *J* = 7.05 Hz, OCOCHCH_2_CH_3_-6’), 1.11 (3H, d, *J* = 6.95 Hz, OCOCHCH_2_CH_3_-7’), 0.94 (3H, t, *J*_1_ = 7.45 Hz, *J*_2_ = 7.40 Hz, OCOCHCH_2_CH_3_-9’), 0.88 (3H, t, *J* = 7.45 Hz, OCOCHCH_2_CH_3_-10’). ^13^C NMR (CDCl_3_, 500 MHz) δ 175.19 (C, C-1’), 175.14 (C, C-3’), 170.58 (C, C-16), 168.82 (C, C-12), 147.06 (C, C-10), 143.59 (C, C-4), 135.42 (C, C-11), 123.80 (CH_2_, C-13), 114.70 (CH_2_, C-14), 114.26 (CH_2_, C-15), 78.92 (CH, C-9), 77.69 (CH, C-6), 74.49 (CH, C-3), 73.96 (CH, C-8), 48.95 (CH, C-5), 46.27 (CH, OCOCH-4’), 41.36 (CH, C-7), 41.14 (CH, OCOCH-2’), 40.57 (CH, C-1), 36.20 (CH_2_, C-2), 26.48 (CH_2_, OCOCHCH_2_-5’), 26.03 (CH_2_, OCOCHCH_2_-8’), 21.21 (CH_3_, OCOCH_3_-17), 16.69 (CH_3_, OCOCHCH_2_CH_3_-6’), 15.78 (CH_3_, OCOCHCH_2_CH_3_-7’), 11.80 (CH_3_, OCOCHCH_2_CH_3_-9’), 11.41 (CH_3_, OCOCHCH_2_CH_3_-10’); (+) EIMS (*m*/*z*) 511.3 (MNa^+^), calculated for C_27_H_36_NaO_8_, 511.2.

Compound **5.** Yield: 15 mg (43%). Colorless needless (CHCl_3_); UV (MeOH) λmax (log ε): 210 (2.90) nm. ^1^H-NMR (CDCl_3_, 500 MHz) δ 6.26 (1H, d, *J* = 3.45 Hz, H-13a), 5.65 (1H, d, *J* = 3.05 Hz, H-13b), 5.56 (1H, m, H-3), 5.52 (1H, t, *J*_1_ = 1.70 Hz, *J*_2_ = 1.8 Hz, H-14a), 5.36 (1H, t, *J*_1_ = 1.70 Hz, *J*_2_ = 1.75 Hz, H-14b), 5.14 (1H, s, H-15a), 5.02 (1H, m, H-8), 4.97 (1H, s, H-15b), 4.15 (1H, ddd, *J* = 9.0, 1.60, 9.0 Hz, H-6), 3.12 (1H, m, H-7), 3.01 (1H, m, H-5), 2.84 (1H, m, H-1), 2.67 (1H, dd, *J* = 5.25 Hz, H-2a), 2.37 (2H, m, H-9a, 9b), 2.16 (3H, s, OCOCH_3_-2’), 2.09 (3H, s, OCOCH_3_-17), 1.79 (1H, m, H-2b). ^13^C-NMR (CDCl_3_, 500 MHz) δ 171.02 (C, C-1’), 170.35 (C, C-16), 169.25 (C, C-12), 147.32 (C, C-4), 141.47 (C, C-10), 137.49 (C, C-11), 122.90 (CH_2_, C-13), 118.66 (CH_2_, C-15), 116.25 (CH_2_, C-14), 78.28 (CH, C-6), 74.82 (CH, C-3), 74.04 (CHOH, C-8), 51.81 (CH, C-5), 47.66 (CH, C-7), 45.68 (CH, C-1), 37.73 (CH_2_, C-2), 36.48 (CH_2_, OCOCH_3_-2’), 21.50 (CH_3_, OCOCH_3_-17)_,_ 21.43 (CH_3_, OCOCH_3_-17); (+) EIMS (*m*/*z*) 369.3 (MNa^+^), calculated for C_19_H_22_NaO_6_, 369.1.

Compound **6.** Yield: 20 mg (53%). Colorless needless (CHCl_3_); UV (MeOH) λmax (log ε): 210 (2.90) nm. ^1^H-NMR (CDCl_3_, 500 MHz) δ 6.23 (1H, d, *J* = 3.45 Hz, H-13a), 5.61 (1H, d, *J* = 3.05 Hz, H-13b), 5.55 (1H, m, H-3), 5.52 (1H, t, *J* = 1.75 Hz, H-14a), 5.35 (1H, t, *J* = 1.65 Hz, H-14b), 5.13 (1H, s, H-15a), 5.03 (1H, m, H-8), 4.92 (1H, d, *J* = 1.25 Hz, H-15b), 4.16 (1H, dt, *J*_1_ = 9 Hz, *J*_2_ =1.6 Hz, H-6), 3.13 (1H, m, H-7), 3.00 (1H, m, H-5), 2.82 (1H, m, H-1), 2.62 (2H, m, H-2a), 2.34 (2H, m, H-9a, 9b), 2.08 (3H, s, OCOCH_3_-17), 1.78 (1H, m, H-2b, OCOCH-2’), 1.24 (3H, d, *J* = 7.10 Hz, OCOCH3-3’), 1.22 (3H, d, *J* = 7.05 Hz, OCOCH3-4’). ^13^C-NMR (CDCl_3_, 500 MHz) δ 176.16 (C, C-1’), 170.81 (C, C-16), 169.02 (C, C-12), 147.10 (C, C-4), 141.31 (C, C-10), 137.55 (C, C-11), 122.45 (CH_2_, C-13), 118.47(CH_2_, C-15), 116.28 (CH_2_, C-14), 77.92 (CH, C-6), 74.60 (CH, C-3), 73.41 (CHOH, C-8), 51.84 (CH, C-5), 47.53 (CH, C-7), 45.66 (CH, C-1), 36.89 (CH_2_, C-9), 36.30 (CH_2_, C-2), 34.25 (CH, OCOCH-2’), 21.28 (CH_3_, OCOCHCH_3_-17)_,_ 19.06 (CH_3_, OCOCHCH_3_-3’), 18.76 (CH_3_, OCOCHCH_3_-4’); (+) EIMS (*m*/*z*) 397.9 (MNa^+^), calculated for C_21_H_26_NaO_6_, 397.2.

Compound **7**. Yield: 15 mg (37%). Colorless needless (CHCl_3_); UV (MeOH) λmax (log ε): 210 (2.90) nm. ^1^H-NMR (CDCl_3_, 500 MHz) δ 6.24 (1H, d, *J* = 3.45 Hz, H-13a), 5.64 (1H, d, *J* = 3.05 Hz, H-13b), 5.56 (1H, m, H-3), 5.53 (1H, t, *J*_1_ = 1.75 Hz, *J*_2_ = 1.95 Hz, H-14a), 5.35 (1H, t, *J*_1_ = 1.75 Hz, *J*_2_ = 1.95 Hz, H-14b), 5.13 (1H, s, H-15a), 5.04 (1H, m, H-8), 4.95 (1H, s, H-15b), 4.15 (1H, dt, *J* = 9.05, 1.55, 9.05 Hz, H-6), 3.12 (1H, m, H-7), 2.83 (1H, m, H-1), 2.67 (1H, dd, *J*_1_ = 5.25 Hz, H-2a), 2.38 (1H, m, H-9a), 2.33 (1H, m, H-9b), 2.27 (2H, m, OCOCH_2_-2’), 2.17 (1H, m, OCOCH_2_CH-3’), 2.09 (3H, s, OCOCH_3_-17), 1.78 (1H, m, H-2b), 1.02 (3H, d, *J* = 1.5 Hz, OCOCH_2_CHCH_3_-4’), 1.00 (3H, d, *J* = 1.5 Hz, OCOCH_2_CHCH_3_-5’). ^13^C-NMR (CDCl_3_, 500 MHz) δ 172.28 (C, C-1’), 170.81 (C, C-16), 169.05 (C, C-12), 147.11 (C, C-4), 141.31 (C, C-10), 137.38 (C, C-11), 122.62 (CH_2_, C-13), 118.41(CH_2_, C-15), 116.09 (CH_2_, C-14), 78.05 (CH, C-6), 74.60 (CH, C-3), 73.48 (CH, C-8), 51.65 (CH, C-5), 47.47 (CH, C-7), 45.54 (CH, C-1), 43.56 (CH, OCOCH_2_-2’), 37.41(CH_2_, C-9), 36.28 (CH_2_, C-2), 25.57 (CH, OCOCH_2_CH-3’), 22.44 (CH_3_, OCOCH_3_-17), 21.28 (CH_3_, OCOCH_2_CHCH_3_-4’),18.44 (CH_3_, OCOCH_2_CHCH_3_-5’); (+) EIMS (*m*/*z*) 411.0 (MNa^+^), calculated for C_22_H_28_NaO_6_, 411.2.

Compound **8.** Yield: 10 mg (26%). Colorless needless (CHCl_3_); UV (MeOH) λmax (log ε): 210 (2.90) nm. ^1^H-NMR (CDCl_3_, 500 MHz) δ 6.24 (1H, d, *J* = 3.4 Hz, H-13a), 5.64 (1H, d, *J* = 3.05 Hz, H-13b), 5.56 (1H, m, H-3), 5.53 (1H, t, *J*_1_ = 1.75 Hz, *J*_2_ = 1.95 Hz, H-14a), 5.36 (1H, t, *J*_1_ = 1.55 Hz, *J*_2_ = 1.60 Hz, H-14b), 5.13 (1H, s, H-15a), 5.05 (1H, m, H-8), 4.93 (1H, s, H-15b), 4.16 (1H, dt, *J* = 9 Hz, H-6), 3.13 (1H, m, H-7), 3.01 (1H, m, H-5), 2.83 (1H, m, H-1), 2.64 (1H, dd, *J* = 5.25 Hz, H-2a), 2.44 (1H, m, H-9a), 2.37 (1H, m, H-9b), 2.33 (1H, m, OCOCH-2’), 2.09 (3H, s, OCOCH_3_-17), 1.77 (2H, m, OCOCHCH_2_-3’), 1.51 (1H, m, H-2b), 1.22 (3H, d, *J* = 7.05 Hz, OCOCHCH_3_-5’), 0.96 (3H, t, *J* = 7.4 Hz, OCOCHCH_2_CH_3_-4’). (+) EIMS (*m*/*z*) 411.0 (MNa^+^), calculated for C_22_H_28_NaO_6_, 411.2.

### 4.5. Cell Culture and Treatment

The human colorectal cell line HCT116 was obtained from the American Tissue Culture Collection, ATCC, Manassas, Virginia. The HCT116 p53^−/−^ cells was provided byCarlos Maria Galmarini, PharmaMar, Madrid, Spain. The NCM460 normal-like cell line was purchased from INCELL Corporation, LLC San Antonio, Texas. The human colorectal cell line HCT116 was cultured in RPMI medium supplemented with 10% fetal bovine serum, 1% sodium pyruvate, and 1% penicillin-streptomycin. HCT116 p53^−/−^ cells were cultured in DMEM medium containing 10% fetal bovine serum, 1% sodium pyruvate, 1% nonessential amino acids, and 1% penicillin-streptomycin. The NCM460 normal-like cell line was cultured in M3: Base medium (INCELL Corporation, LLC) supplemented with 10% fetal bovine serum. All cells were maintained in a humidified incubator at 37 °C, 95% air, and 5% CO_2_.

5-FU (Sigma) was dissolved in DMSO at 0.5 mM concentration and stored at −20 °C. Cells in logarithmic phase were seeded in 96-well plates. When 40–50% confluent, cells were treated with vehicle solvent (DMSO) as control or with increasing concentrations of the tested compounds (1, 2.5, 5, 10, 15, 25, and 100 µg/mL) up to 48 h. The use of high concentrations of the tested compounds was mandatory for the calculation of the IC_50_ values. The highest used concentrations for the solvent controls did not exceed 1% DMSO; this concentration did not affect the viability of the tested cell lines.

### 4.6. Cytotoxicity and Growth Assays

The cytotoxicity of the tested compounds was quantitatively assessed by the Cytotoxicity Detection Kit^PLUS^ (LDH) (Sigma-Aldrich, Taufkirchen, Germany) at 6 h post-treatment. This kit measures the activity of the stable cytosolic lactate dehydrogenase (LDH) enzyme released to the outer milieu after lysis of damaged cells. When quantified at short time points, it allows the detection of the acute effect of the drug highlighted by cell bursting and LDH release. Briefly, cells were seeded in 96-well plates and treated with increasing concentrations of the tested compounds. Six hours post-treatment, 50 μL of the medium and 50 μL of the reaction mix were incubated for 30 min and then stopped by the addition of the stop solution. The absorbance was detected at 490 nm with the use of an ELISA microplate reader. The results are representative of three independent experiments done in triplicate wells expressed as percentage of control cells and plotted as the mean ± SEM.

Cell growth was assessed by the use of the thiazolyl blue tetrazolium bromide (MTT) assay (Sigma M5655). It quantifies the metabolic activity of the mitochondria converting tetrazolium salt into a blue formazan crystal. Briefly, exponentially growing cells were seeded into 96-well plates and treated with the indicated concentrations of the compounds up to 48 h. At each time point, the resulting formazan crystals were dissolved in a solubilization solution (6 M HCl, 10% SDS, and 5% isobutanol). The absorbance was recorded at 595 nm using an ELISA microplate reader. Cell growth was assayed in triplicates. The results are expressed as percentage of control and represent the average of three independent experiments ± SEM. Cell viability was confirmed by the trypan blue dye exclusion assay.

### 4.7. Statistical Analysis

Data are expressed as mean ± standard error of the mean (SEM) of three independent experiments. Results were analyzed by analysis of variance ANOVA and Bonferroni as post hoc using GraphPad Prism 7. The exact IC_50_ values at 48 h were calculated using GraphPad Prism 7 software ± standard error. Differences were considered significant if *p* < 0.05. Approximate IC_50_ values for Sal-**A** were calculated as 100% cell death was not achieved at 100 µg/mL. Higher Sal-**A** concentrations than 100 µg/mL were not possible due to solubility limitations.

## Figures and Tables

**Table 1:** IC_50_ values of Sal-**A,** Sal-**B,** their C8-OH and C9-OH modified derivatives, and 5-Fluorouracil (5-FU). The IC_50_ values represent the concentrations that cause 50% inhibition of cell growth at 48 h. Cells were treated with the different compounds for 48 h. All data (mean ± SEM) are the average of three independent experiments performed in triplicate measurements. Cancer cell lines: HCT116 (human colorectal cancer wild type for p53), HCT116 p53^−/−^ (human colorectal cancer null for p53); NCM460 (normal-like human colon mucosa cell with wild-type p53). ND: not detected. * Approximate IC_50_ values. (Approximate IC_50_ values for Sal-**A** and 5-FU were calculated as 100% cell death was not achieved at 100 µg/mL).

Compounds	IC_50_ Values ± SEM					
	HCT116		HCT116 p53^−/−^		NCM460	
	µg/mL	µM	µg/mL	µM	µg/mL	µM
A	31.9 *	99.6 *	69.5 *	217.1 *	ND	ND
1	2.9 ± 0.44	6.8 ± 1.03	2.8 ± 0.68	6.6 ± 1.59	ND	ND
2	2.3 ± 0.13	4.8 ± 0.27	1.8 ± 0.09	3.7 ± 0.19	6.9 ± 1.52	14.3 ± 3.15
3	6.9 ± 1.90	13.5 ± 3.72	7.2 ± 1.05	14.1 ± 2.05	ND	ND
4	1.7 ± 0.34	3.3 ± 0.67	2.2 ± 0.18	4.3 ± 0.35	10.2 ± 0.54	19.9 ± 1.06
						
B	4.4 ± 0.89	14.4 ± 2.92	5.1 ± 2.29	16.7 ± 7.51	21.8 ± 4.09	71.5 ± 13.40
5	2.3 ± 0.52	6.2 ± 1.41	2.1 ± 0.23	5.7 ± 0.62	ND	ND
6	1.5 ± 0.18	3.8 ± 0.45	2.1 ± 0.16	5.3 ± 0.40	5.2 ± 0.55	13.1 ± 1.38
7	3.8 ± 0.27	9.3 ± 0.66	3.8 ± 0.78	9.3 ± 1.90	ND	ND
8	2.5 ± 0.45	6.1 ± 1.09	1.9 ± 0.50	4.6 ± 1.22	7.1 ± 0.66	17.3 ± 1.61
						
5-FU	2.5 *	19.2 *	2.5 *	19.2 *	ND	ND

## Data Availability

The data presented in this study are available in the paper or supplementary material.

## Supplementary Materials

The following are available online. Figure S1: Effect of Sal-A and Sal-B on the growth of HCT116 colorectal cancer cells. Figure S2: Effect of Sal-A and Sal-B on the growth of HCT116 p53-/- colorectal cancer cells. Figure S3: Effect of the different Sal-A derivatives on the growth of HCT116 cells. Figure S4: Effect of the different Sal-B derivatives on the growth of HCT116 cells. Figure S5: Effect of the different Sal-A derivatives on the growth of HCT116 p53-/- cells. Figure S6: Effect of the different Sal-B derivatives on the growth of HCT116 p53-/- cells. Figure S7: Effect of Sal-A and its most potent derivatives on the growth of NCM460 cells. Figure S8: Effect of Sal-B and its most potent derivatives on the growth of NCM460 cells. Figure S9: Cytotoxic effect of Sal-A and Sal-B on HCT116 cells. Figure S10: Cytotoxic effect of Sal-A derivatives on HCT116 cells. Figure S11: Cytotoxic effect of Sal-B derivatives on HCT116 cells. Figure S12: ^1^H NMR spectrum of compound 1 (CDCl3, 500 MHz.) Figure S13: ^13^C NMR spectrum of compound 1 at 20 K (CDCl3, 500 MHz) Figure S14: ^1^H NMR spectrum of compound 2 (CDCl3, 500 MHz) Figure S15: 13C NMR spectrum of compound 2 at 20 K (CDCl3, 500 MHz). Figure S16: ^1^H NMR spectrum of compound 3 (CDCl3, 500 MHz) Figure S17: ^13^C NMR spectrum of compound 3 at 20 K. Figure S18: ^1^H NMR spectrum of compound 4 (CDCl3, 500 MHz). Figure S19: ^13^C NMR spectrum of compound 4 at 20 K (CDCl3, 500 MHz). Figure S20: ^1^H NMR spectrum of compound 5 (CDCl3, 500 MHz). Figure S21: ^13^C NMR spectrum of compound 5 at 20 K (CDCl3, 500 MHz). Figure S22: ^1^H NMR spectrum of compound 6 (CDCl3, 500 MHz). Figure S23: ^13^C NMR spectrum of compound 6 at 20 K (CDCl3, 500 MHz). Figure S24: ^1^H NMR spectrum of compound 7 (CDCl3, 500 MHz). Figure S25: ^13^C NMR spectrum of compound 7 at 20 K (CDCl3, 500 MHz). Figure S26: ^1^H NMR spectrum of compound 8 (CDCl3, 500 MHz).

## Sample Availability

Samples of the compounds Sal-**A**, Sal-**B** and derivatives 1 to 8are available from the authors.
