# Supplementary material for: The Effect of Different Ester Chain Modifications of Two Guaianolides for Inhibition of Colorectal Cancer Cell Growth
Source: Molecules. 2021 Sep 9;26(18):5481. doi: 10.3390/molecules26185481 (PMC8469367; doi:10.3390/molecules26185481)
Supplement: Supplementary file 1 [file molecules-26-05481-s001.zip › molecules-1333051-supplementary.pdf]

SUPPORTING INFORMATION

The Effect of Different Ester Chain Modifications of Two Guaianolides for Inhibition of Colorectal Cancer Cell Growth

Lamis Al Aaraj [1,2], Berthe Hayar [1,3] Zaynab Jaber [1,3], Walid Saad [1,4], Najat A. Saliba [1,2], Nadine Darwiche [1,3,*] and Tarek Ghaddar [1,2,*]

1 AUB Nature Conservation Center, American University of Beirut, Beirut P.O. Box 11-0236, Lebanon; lamis.agha86@gmail.com (L.A.A.); bh48@aub.edu.lb (B.H.); Zjaber90@gmail.com (Z.J.); ws20@aub.edu.lb (W.S.); ns30@aub.edu.lb (N.A.S.)
2 Department of Chemistry, American University of Beirut, Beirut P.O. Box 11-0236, Lebanon
3 Department of Biochemistry and Molecular Genetics, American University of Beirut, Beirut P.O. Box 11-0236, Lebanon
4 Department of Chemical Engineering, American University of Beirut, Beirut P.O. Box 11-0236, Lebanon
* Correspondence: nd03@aub.edu.lb (N.D.); tg02@aub.edu.lb (T.G.); Tel.: +961-1-350000 (ext. 4803) (N.D.); +961-1-350000 (ext. 4057) (T.G.); Fax: +961-1-374374 (N.D.); +961-1-365217 (T.G.)

Table of contents

No		Pages
1	**Figure S1:** Effect of Sal-**A** and Sal-**B** on the growth of HCT116 colorectal cancer cells	3
2	**Figure S2:** Effect of Sal-**A** and Sal-**B** on the growth of HCT116 *p53*$^{-/-}$ colorectal cancer cells	3
3	**Figure S3:** Effect of the different Sal-**A** derivatives on the growth of HCT116 cells	4
4	**Figure S4:** Effect of the different Sal-**B** derivatives on the growth of HCT116 cells	5
5	**Figure S5:** Effect of the different Sal-**A** derivatives on the growth of HCT116 *p53*$^{-/-}$ cells	6
6	**Figure S6:** Effect of the different Sal-**B** derivatives on the growth of HCT116 *p53*$^{-/-}$ cells	7
7	**Figure S7:** Effect of Sal-**A** and its most potent derivatives on the growth of NCM460 cells	8
8	**Figure S8:** Effect of Sal-**B** and its most potent derivatives on the growth of NCM460 cells	9
9	**Figure S9:** Cytotoxic effect of Sal-**A** and Sal-**B** on HCT116 cells	10
10	**Figure S10:** Cytotoxic effect of Sal-**A** derivatives on HCT116 cells	11
11	**Figure S11:** Cytotoxic effect of Sal-**B** derivatives on HCT116 cells	12
12	**Figure S12:** ^{1}H NMR spectrum of compound **1** ($CDCl_3$, 500 MHz)	13
13	**Figure S13:** ^{13}C NMR spectrum of compound **1** at 20 K ($CDCl_3$, 500 MHz)	14
14	**Figure S14:** ^{1}H NMR spectrum of compound **2** ($CDCl_3$, 500 MHz)	15

15	**Figure S15:** ^{13}C NMR spectrum of compound **2** at 20 K ($CDCl_3$, 500 MHz)	16
16	**Figure S16:** ^{1}H NMR spectrum of compound **3** ($CDCl_3$, 500 MHz)	17
17	**Figure S17:** ^{13}C NMR spectrum of compound **3** at 20 K	18
18	**Figure S18:** ^{1}H NMR spectrum of compound **4** ($CDCl_3$, 500 MHz)	19
19	**Figure S19:** ^{13}C NMR spectrum of compound **4** at 20 K ($CDCl_3$, 500 MHz)	20
20	**Figure S20:** ^{1}H NMR spectrum of compound **5** ($CDCl_3$, 500 MHz)	21
21	**Figure S21:** ^{13}C NMR spectrum of compound **5** at 20 K ($CDCl_3$, 500 MHz)	22
22	**Figure S22:** ^{1}H NMR spectrum of compound **6** ($CDCl_3$, 500 MHz)	23
23	**Figure S23:** ^{13}C NMR spectrum of compound **6** at 20 K ($CDCl_3$, 500 MHz)	24
24	**Figure S24:** ^{1}H NMR spectrum of compound **7** ($CDCl_3$, 500 MHz)	25
25	**Figure S25:** ^{13}C NMR spectrum of compound **7** at 20 K ($CDCl_3$, 500 MHz)	26
26	**Figure S26:** ^{1}H NMR spectrum of compound **8** ($CDCl_3$, 500 MHz)	27

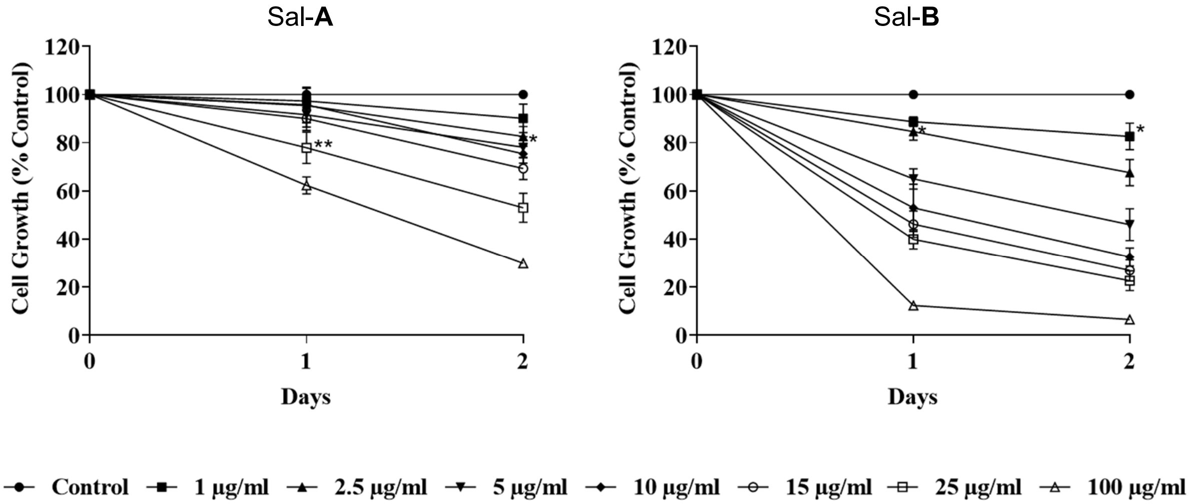

Figure S1. Effect of Sal-**A** and Sal-**B** on the growth of HCT116 colorectal cancer cells. Cells were treated with 1 % DMSO (control) or the indicated concentrations of the compounds for up to two days. Cell growth was determined in triplicate wells using the MTT cell proliferation assay. Results are expressed as percentage of control and represent the average of three independent experiments ± SEM. Statistical significance is reported by two-way ANOVA post hoc Bonferroni's multiple comparisons test indicating differences between treated cells and control at various time points (*, $P < 0.05$; **, $P < 0.01$).

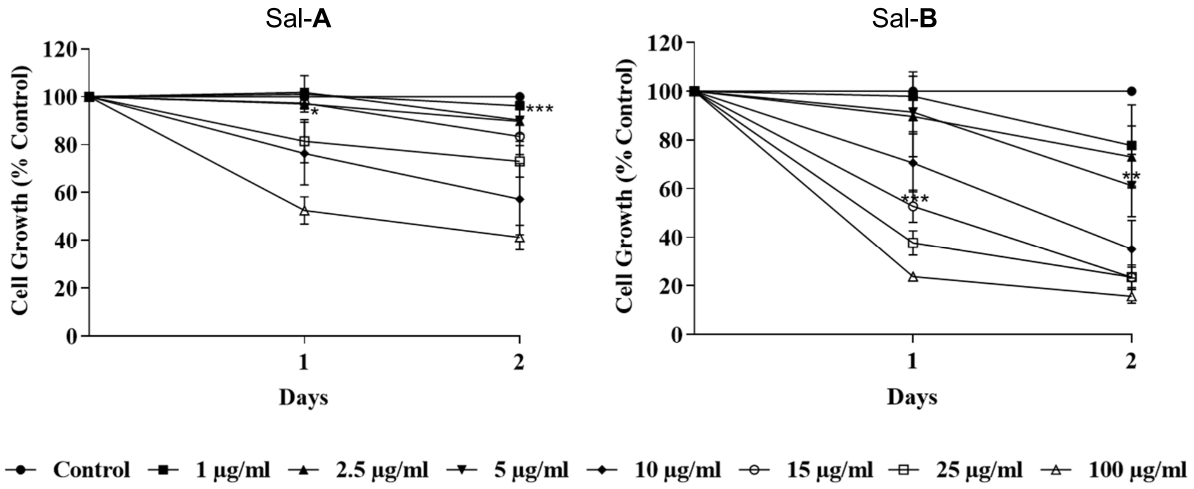

Figure S2. Effect of Sal-**A** and Sal-**B** on the growth of HCT116 *p53*$^{-/-}$ colorectal cancer cells. Cells were treated with 1 % DMSO (control) or the indicated concentrations of the compounds for up to two days. Cell growth was determined in triplicate wells using the MTT cell proliferation assay. Results are expressed as percentage of control and represent the average of three independent experiments ± SEM. Statistical significance is reported by two-way ANOVA post hoc Bonferroni's multiple comparisons test indicating differences between treated cells and control at various time points (*, $P < 0.05$; **, $P < 0.01$; ***, $P < 0.001$).

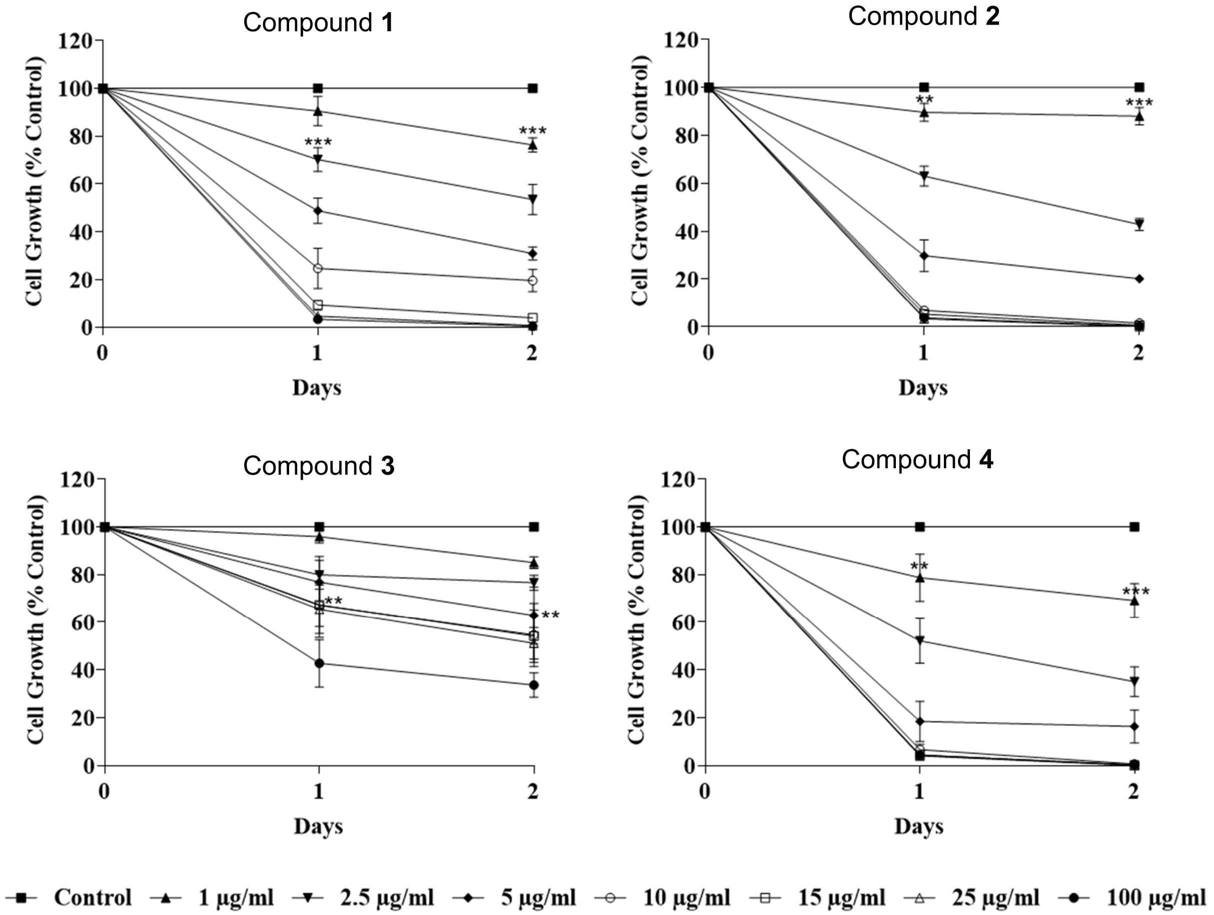

Figure S3. Effect of the different Sal-**A** derivatives with different hydrophobicities on the growth of HCT116 cells. Cells were treated with 1 % DMSO (control) or the indicated concentrations of the compounds for up to two days. Cell growth was determined in triplicate wells using the MTT cell proliferation assay. Results are expressed as percentage of control and represent the average of three independent experiments ± SEM. Statistical significance is reported by two-way ANOVA post hoc Bonferroni's multiple comparisons test indicating differences between treated cells and control at various time points (**, $P < 0.01$; ***, $P < 0.001$).

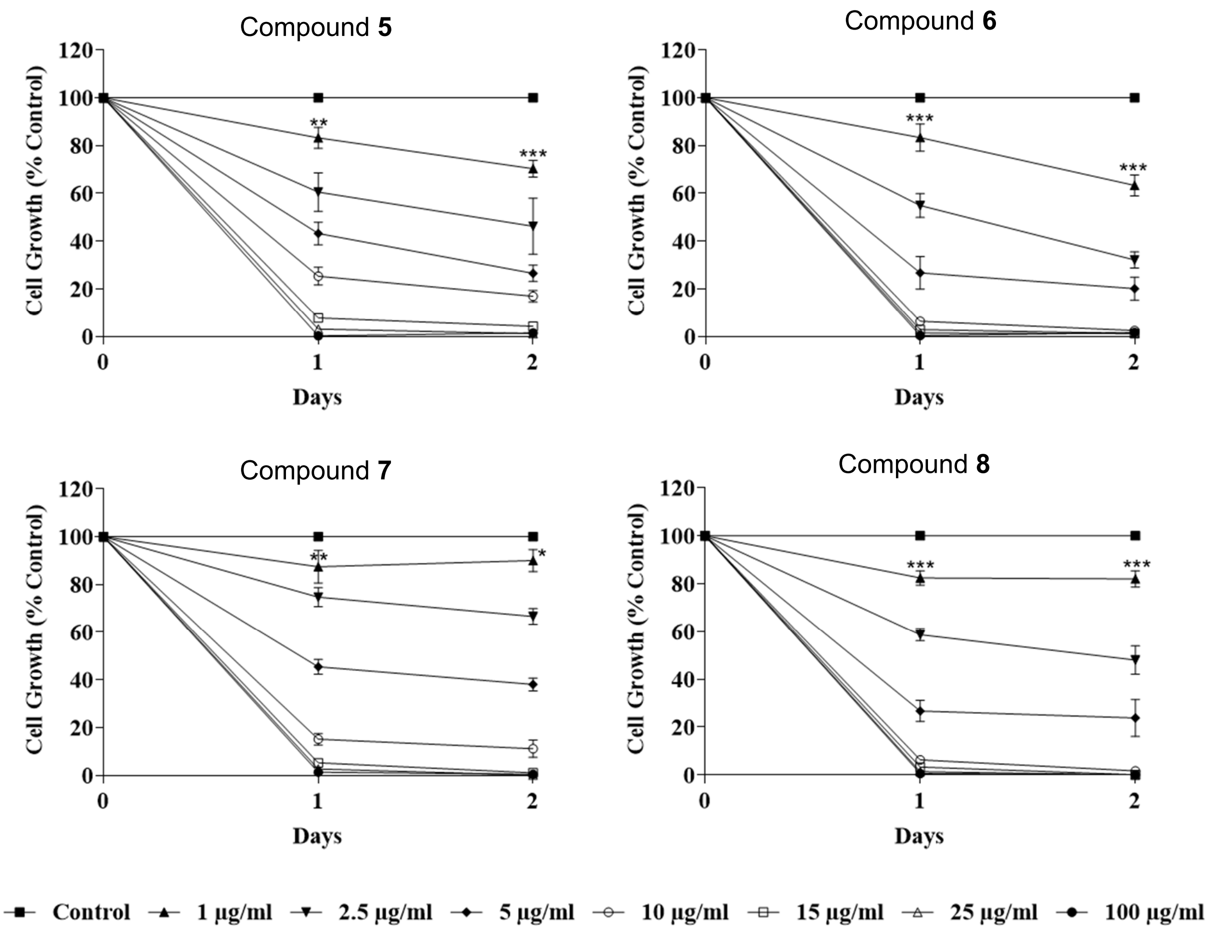

Figure S4. Effect of the different Sal-**B** derivatives with different hydrophobicities on the growth of HCT116 cells. Cells were treated with 1 % DMSO (control) or the indicated concentrations of the compounds for up to two days. Cell growth was determined in triplicate wells using the MTT cell proliferation assay. Results are expressed as percentage of control and represent the average of three independent experiments ± SEM. Statistical significance is reported by two-way ANOVA post hoc Bonferroni's multiple comparisons test indicating differences between treated cells and control at various time points (*, $P < 0.05$; **, $P < 0.01$; ***, $P < 0.001$).

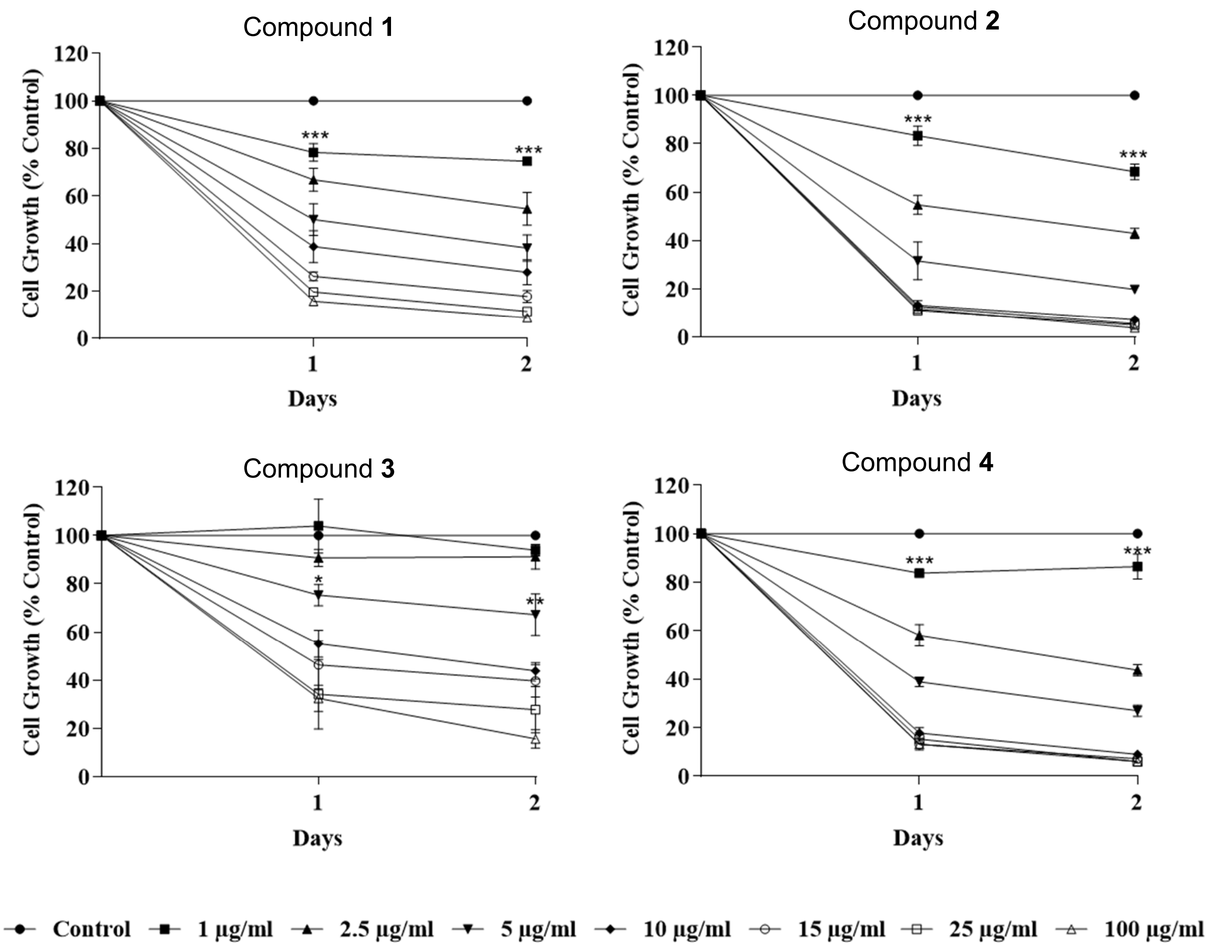

Figure S5. Effect of the different Sal-**A** derivatives with different hydrophobicities on the growth of HCT116 *p53*$^{-/-}$ cells. Cells were treated with 1 % DMSO (control) or the indicated concentrations of the compounds for up to two days. Cell growth was determined in triplicate wells using the MTT cell proliferation assay. Results are expressed as percentage of control and represent the average of three independent experiments ± SEM. Statistical significance is reported by two-way ANOVA post hoc Bonferroni's multiple comparisons test indicating differences between treated cells and control at various time points (*, $P < 0.05$; **, $P < 0.01$; ***, $P < 0.001$).

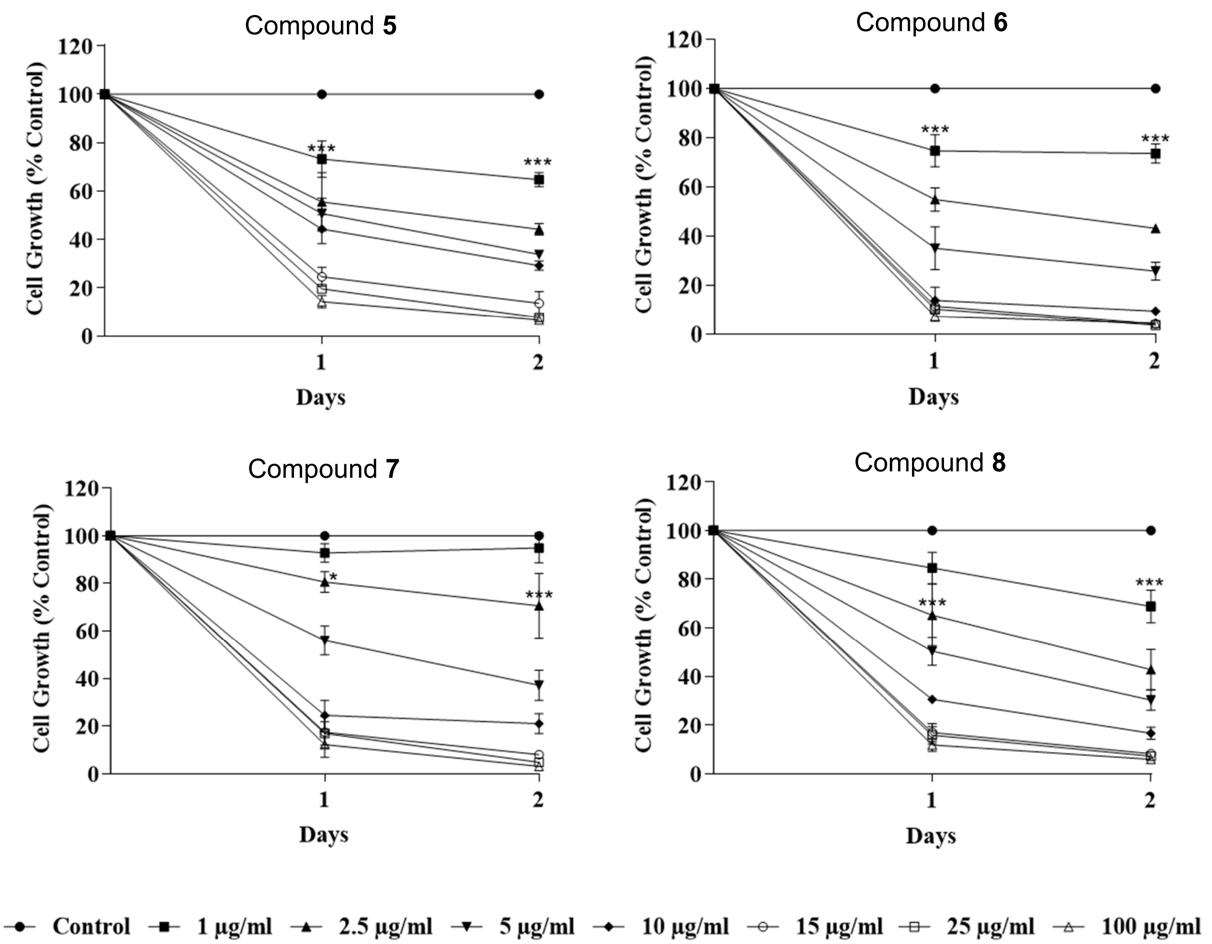

Figure S6. Effect of the different Sal-**B** derivatives with diverse hydrophobicities on the growth of HCT116 *p53*$^{-/-}$ cells. Cells were treated with 1 % DMSO (control) or the indicated concentrations of the compounds for up to two days. Cell growth was determined in triplicate wells using the MTT cell proliferation assay. Results are expressed as percentage of control and represent the average of three independent experiments ± SEM. Statistical significance is reported by two-way ANOVA post hoc Bonferroni's multiple comparisons test indicating differences between treated cells and control at various time points (*, $P < 0.05$; ***, $P < 0.001$).

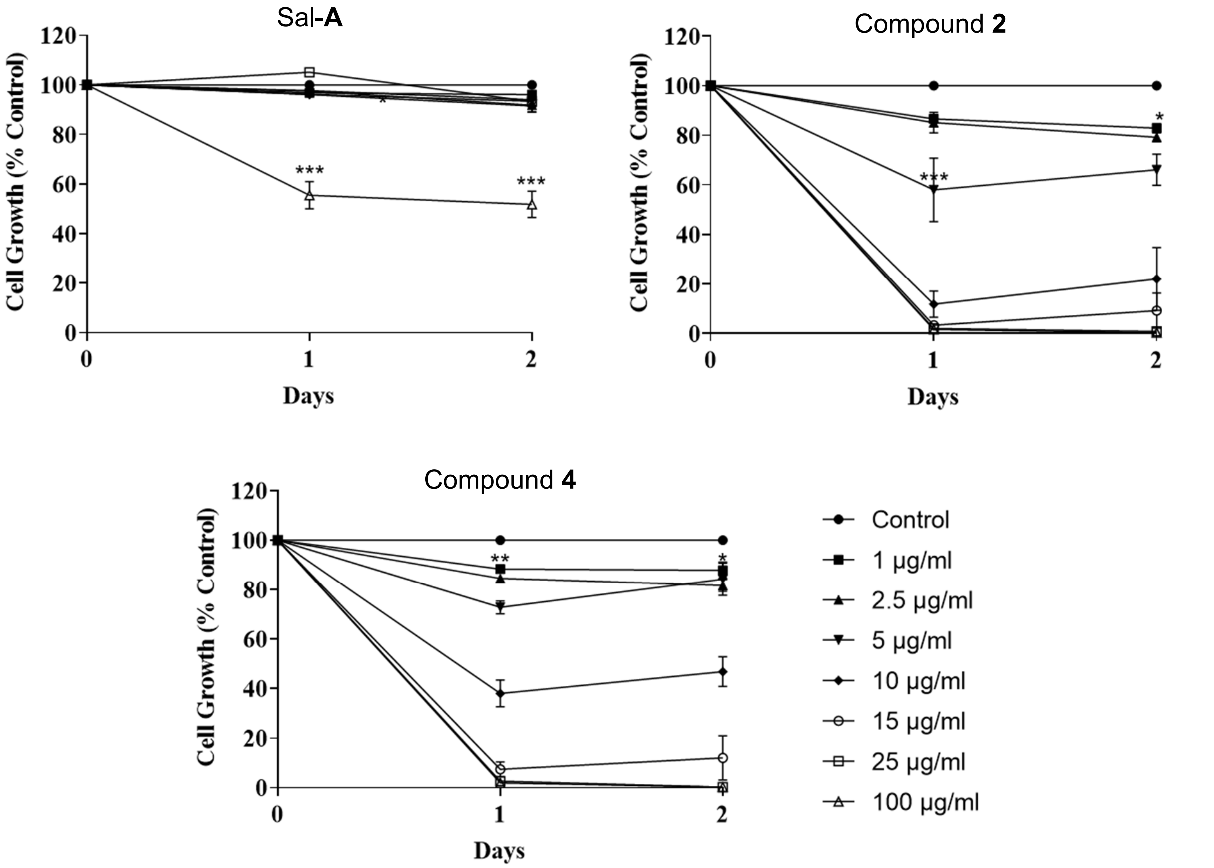

Figure S7. Effect of Sal-**A** and its most potent derivatives on the growth of NCM460 cells. Cells were treated with 1 % DMSO (control) or the indicated concentrations of the compounds for up to two days. Cell growth was determined in triplicate wells using the MTT cell proliferation assay. Results are expressed as percentage of control and represent the average of three independent experiments ± SEM. Statistical significance is reported by two-way ANOVA post hoc Bonferroni's multiple comparisons test indicating differences between treated cells and control at various time points (*, $P < 0.05$; **, $P < 0.01$; ***, $P < 0.001$).

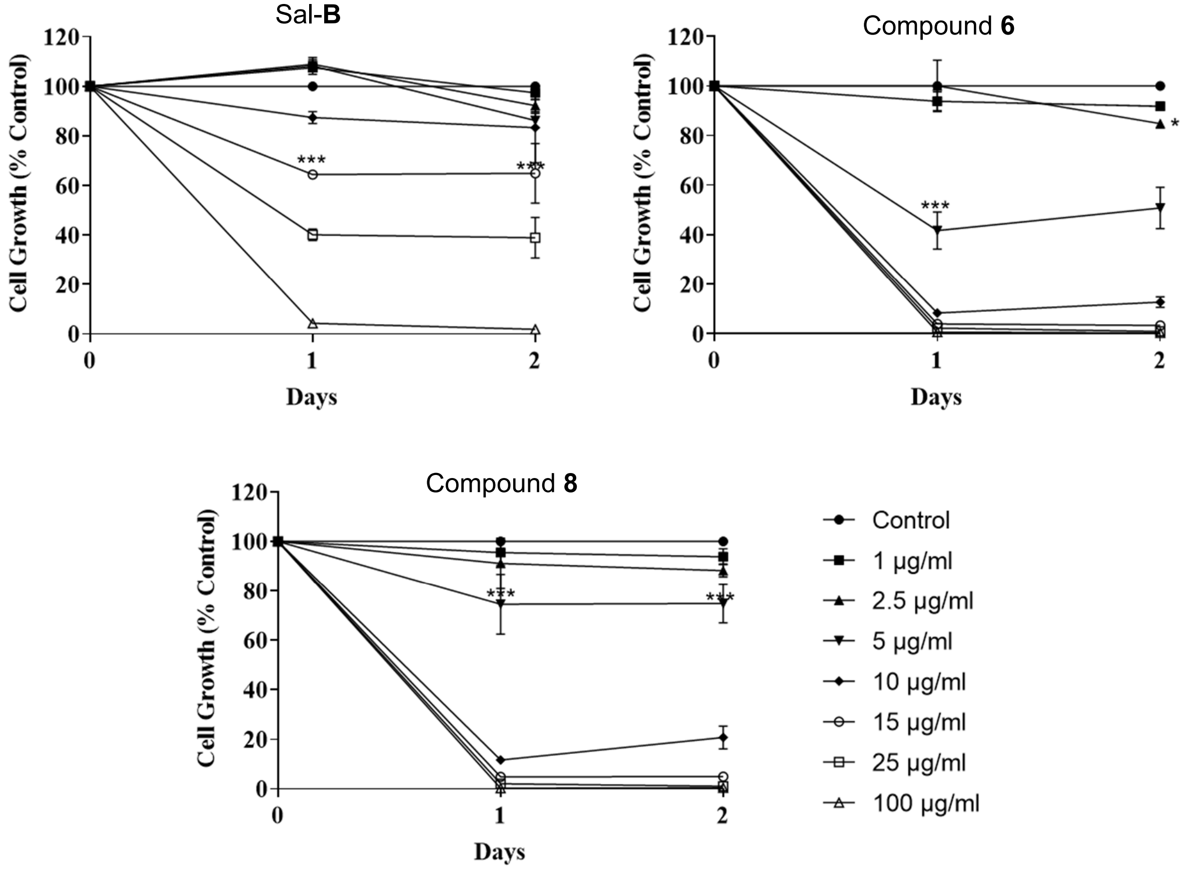

Figure S8. Effect of Sal-**B** and its most potent derivatives on the growth of NCM460 cells. Cells were treated with 1 % DMSO (control) or the indicated concentrations of the compounds for up to two days. Cell growth was determined in triplicate wells using the MTT cell proliferation assay. Results are expressed as percentage of control and represent the average of three independent experiments ± SEM. Statistical significance is reported by two-way ANOVA post hoc Bonferroni's multiple comparisons test indicating differences between treated cells and control at various time points (*, $P < 0.05$; ***, $P < 0.001$).

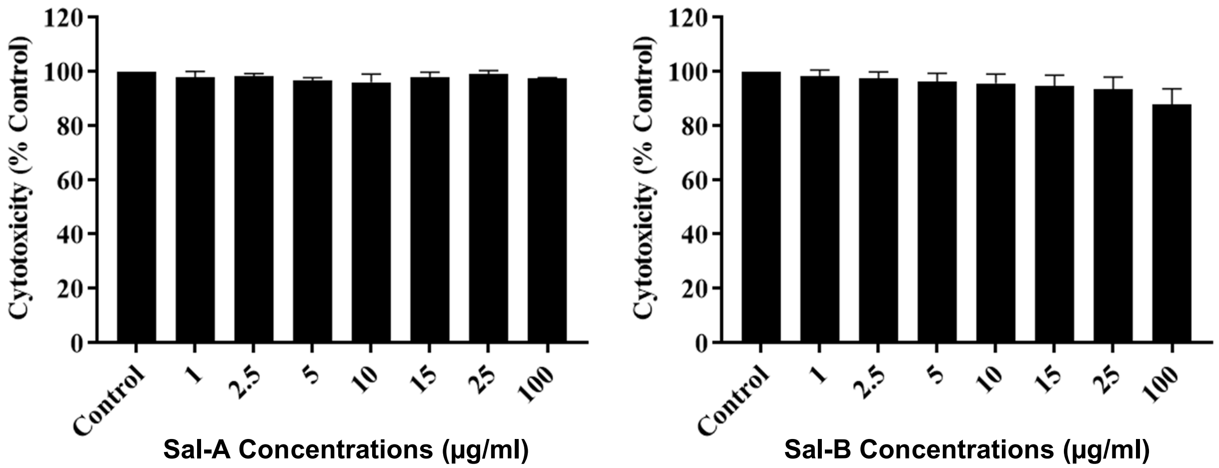

Figure S9. Cytotoxic effect of Sal-**A** and Sal-**B** on HCT116 cells. Cells were treated with 1 % DMSO (control) or the indicated concentrations of the compounds for 6 hours. The cytotoxic activity was determined in triplicate wells by the lactate dehydrogenase assay. Results are expressed as percentage of control and represent the average of three independent experiments ± SEM.

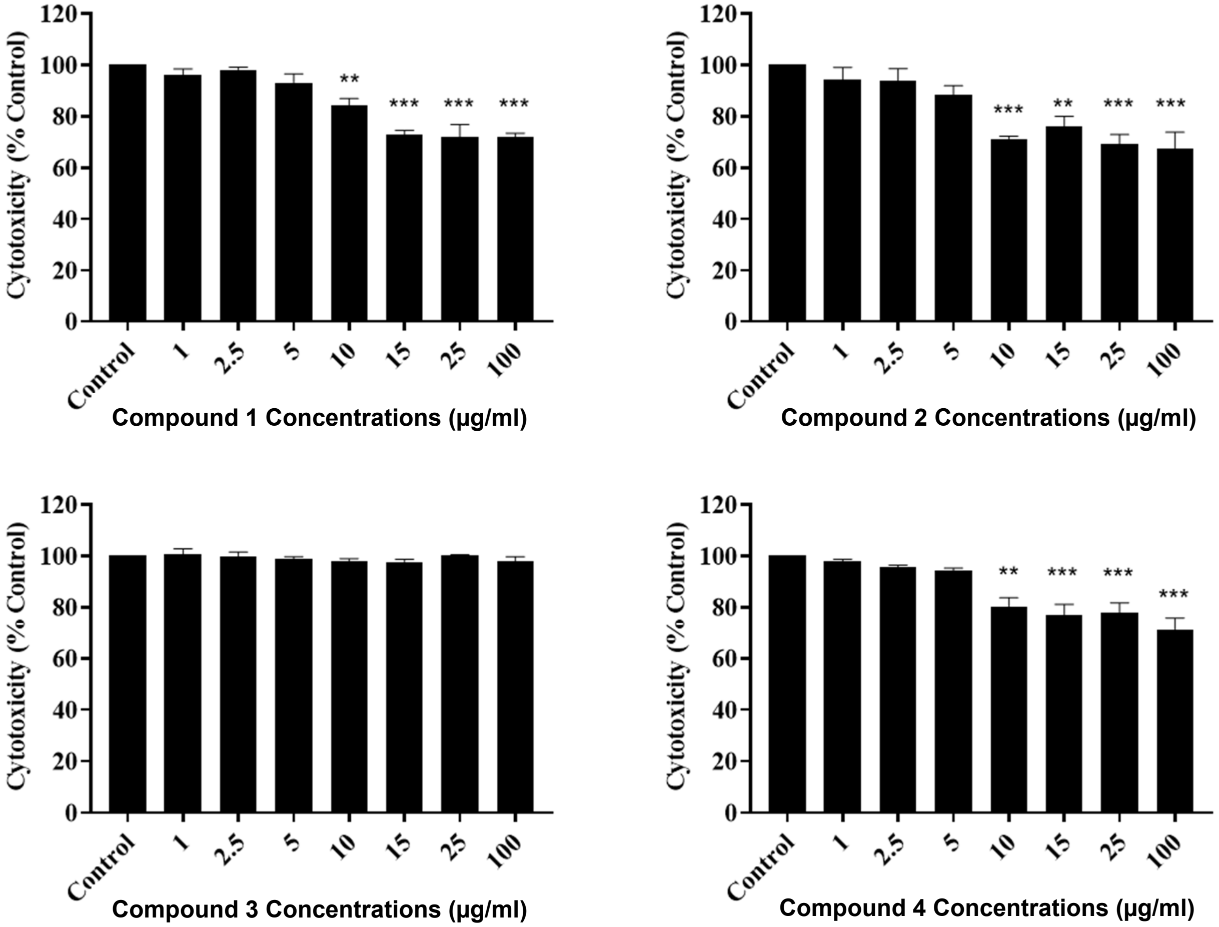

Figure S10. Cytotoxic effect of Sal-**A** derivatives on HCT116 cells. Cells were treated with 1 % DMSO (control) or the indicated concentrations of the compounds for 6 hours. The cytotoxic activity was determined in triplicate wells by the lactate dehydrogenase assay. Results are expressed as percentage of control and represent the average of three independent experiments ± SEM. Statistical significance is reported by one-way ANOVA post hoc Tukey test indicating differences between treatment concentrations and control (**, $P < 0.01$; ***, $P < 0.001$).

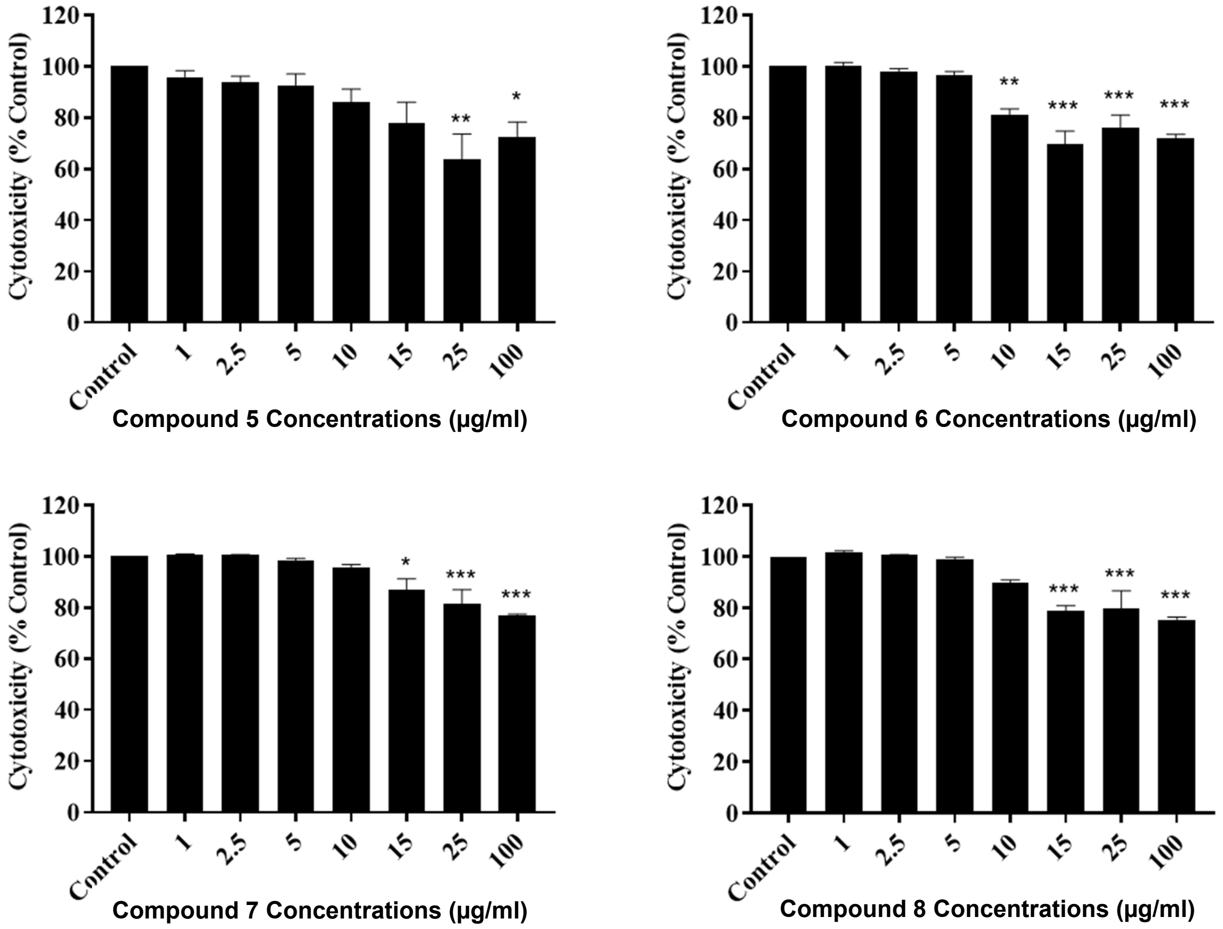

Figure S11. Cytotoxic effect of Sal-**B** derivatives on HCT116 cells. Cells were treated with 1 % DMSO (control) or the indicated concentrations of the compounds for 6 hours. The cytotoxic activity was determined in triplicate wells by the lactate dehydrogenase assay. Results are expressed as percentage of control and represent the average of three independent experiments ± SEM. Statistical significance is reported by one-way ANOVA post hoc Tukey test indicating differences between treatment concentrations and control (*, $P < 0.05$; **, $P < 0.01$; ***, $P < 0.001$).

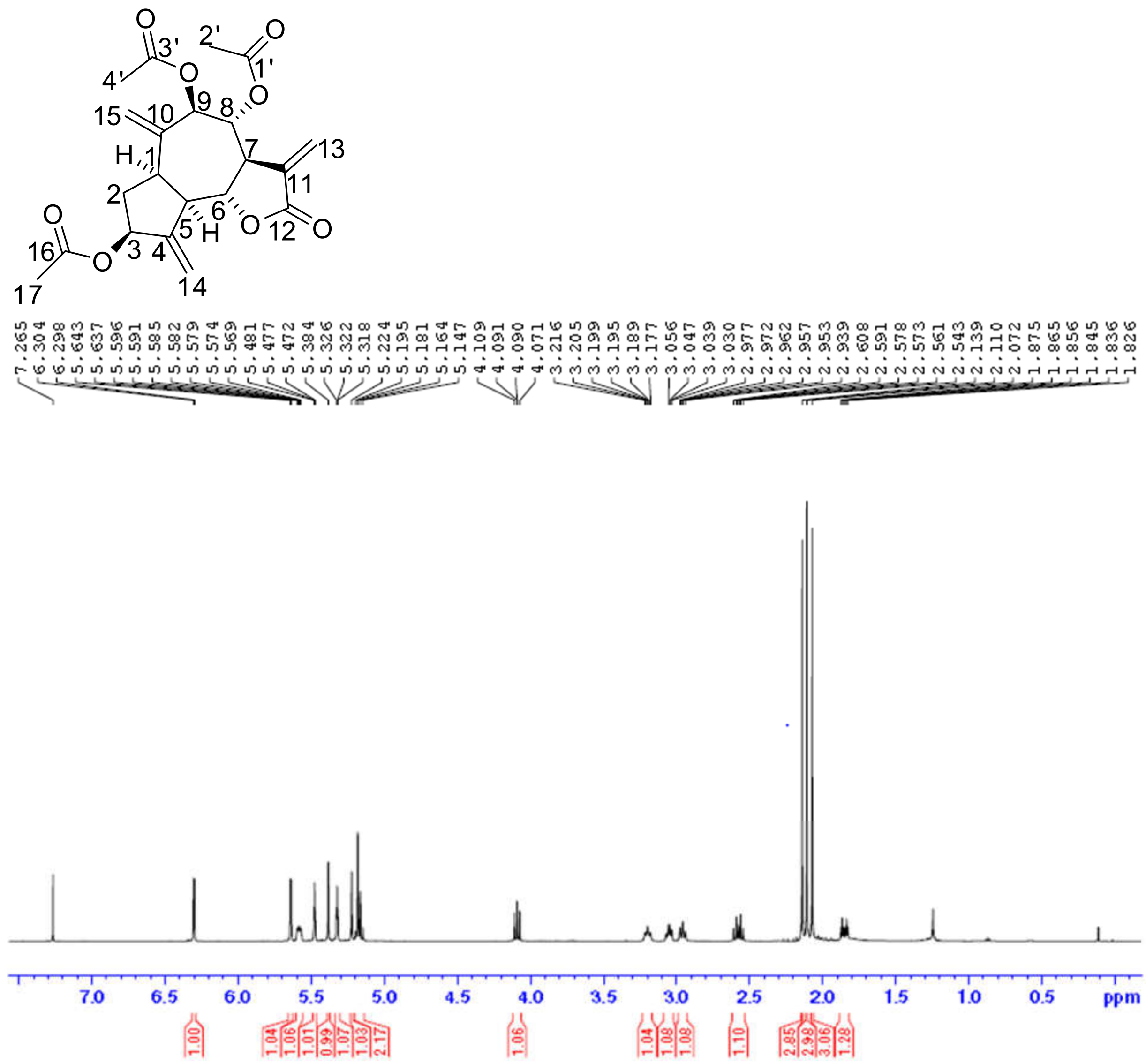

Figure S12: ^{1}H NMR spectrum of compound **1** ($CDCl_3$, 500 MHz).

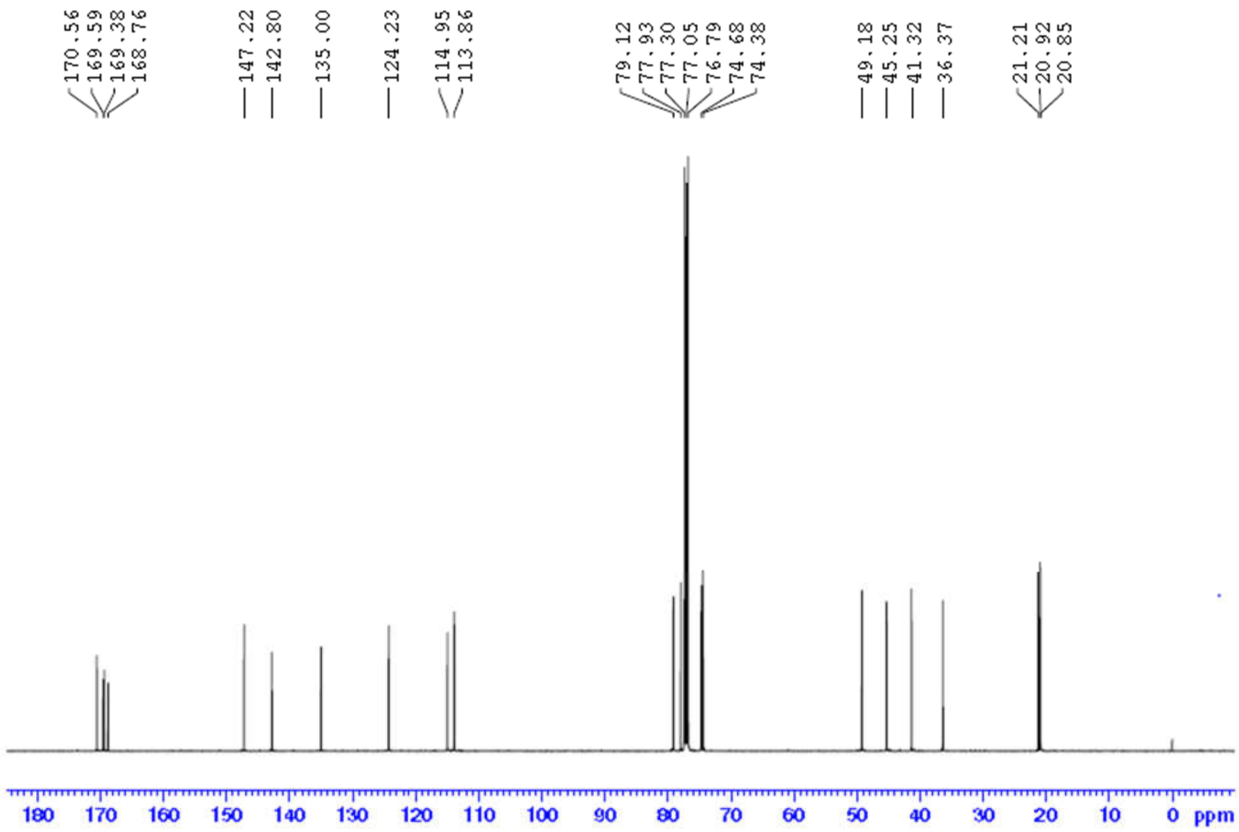

Figure S13: ^{13}C NMR spectrum of compound **1** at 20 K ($CDCl_3$, 500 MHz).

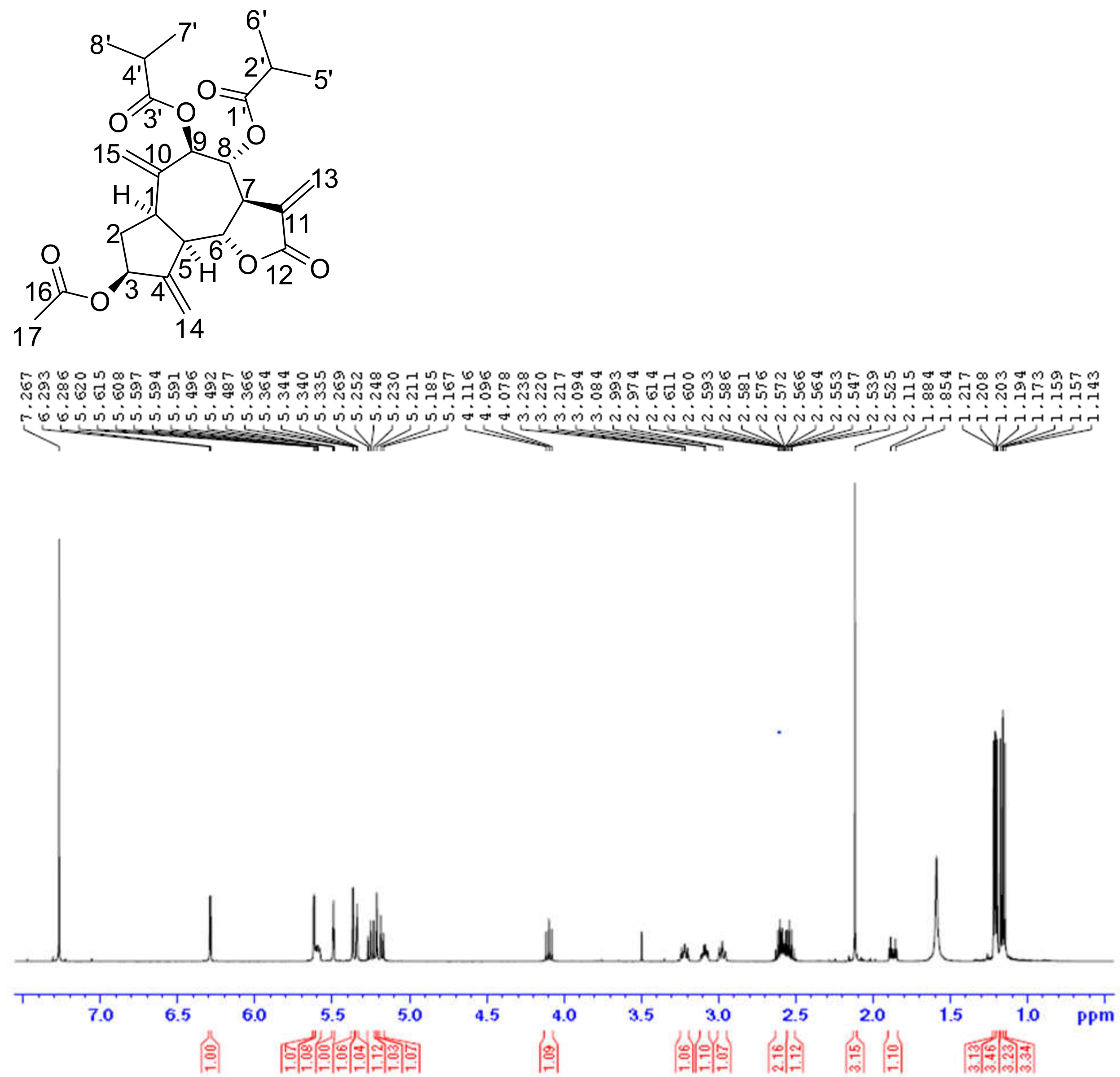

Figure S14: ^{1}H NMR spectrum of compound **2** ($CDCl_3$, 500 MHz).

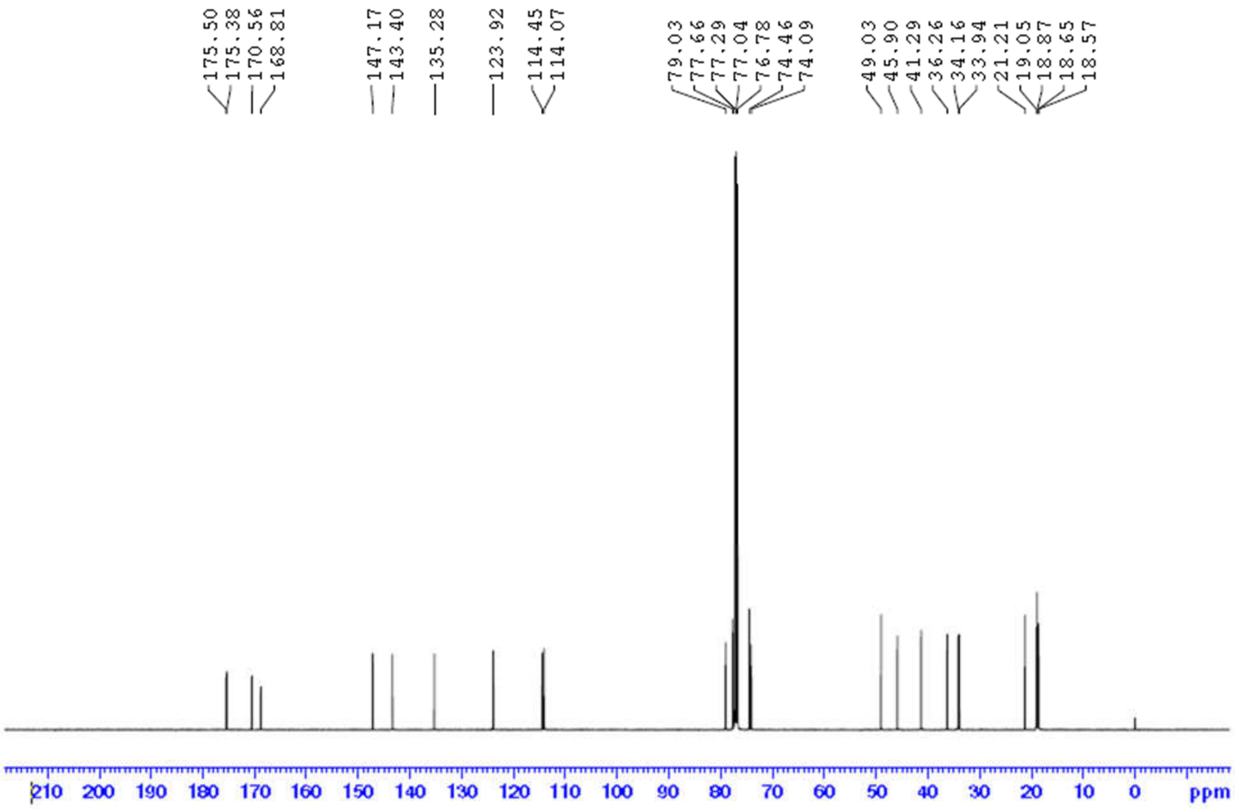

Figure S15: ^{13}C NMR spectrum of compound **2** at 20 K (CDCl3, 500 MHz).

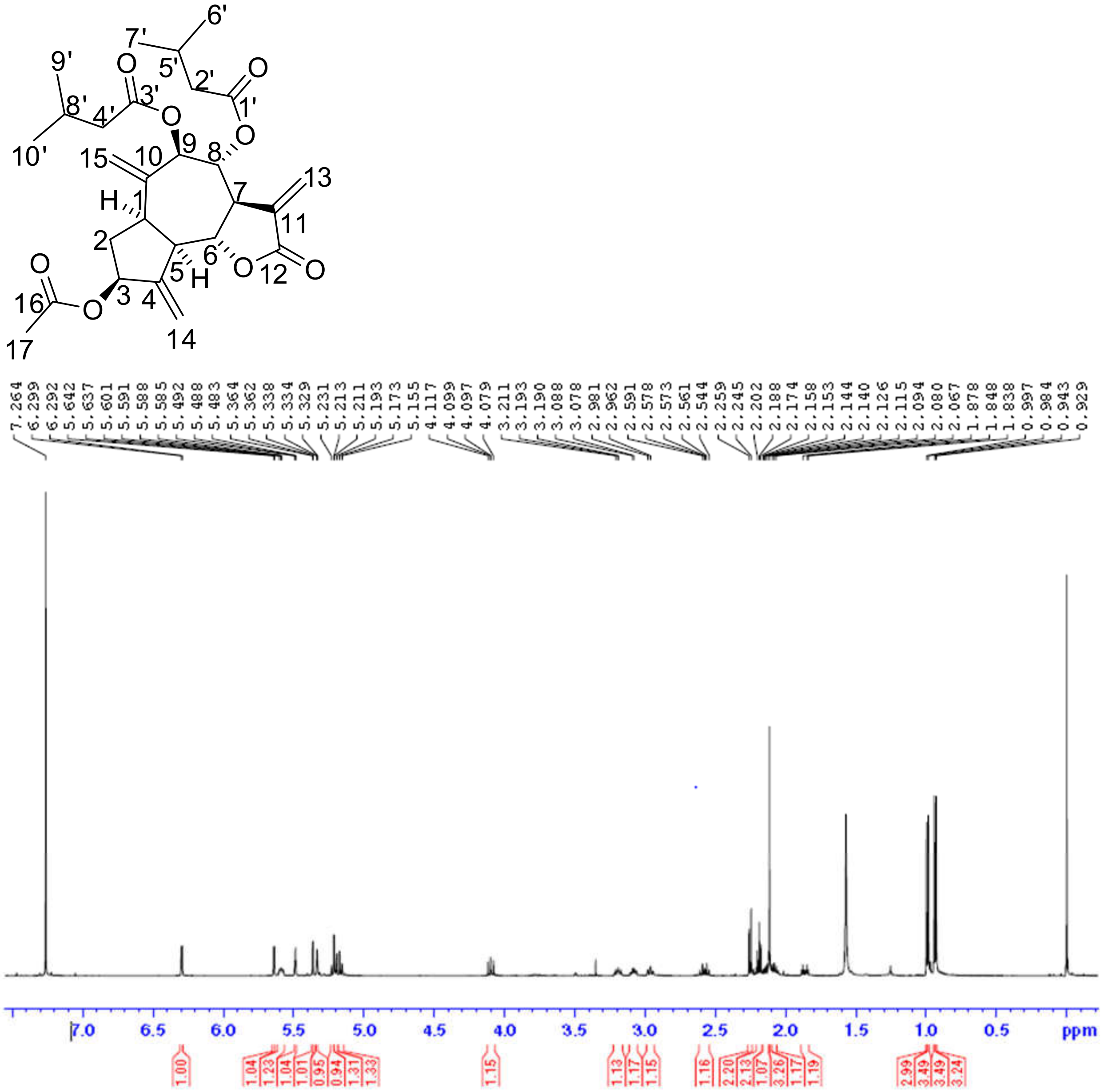

Figure S16: ^{1}H NMR spectrum of compound **3** ($CDCl_3$, 500 MHz).

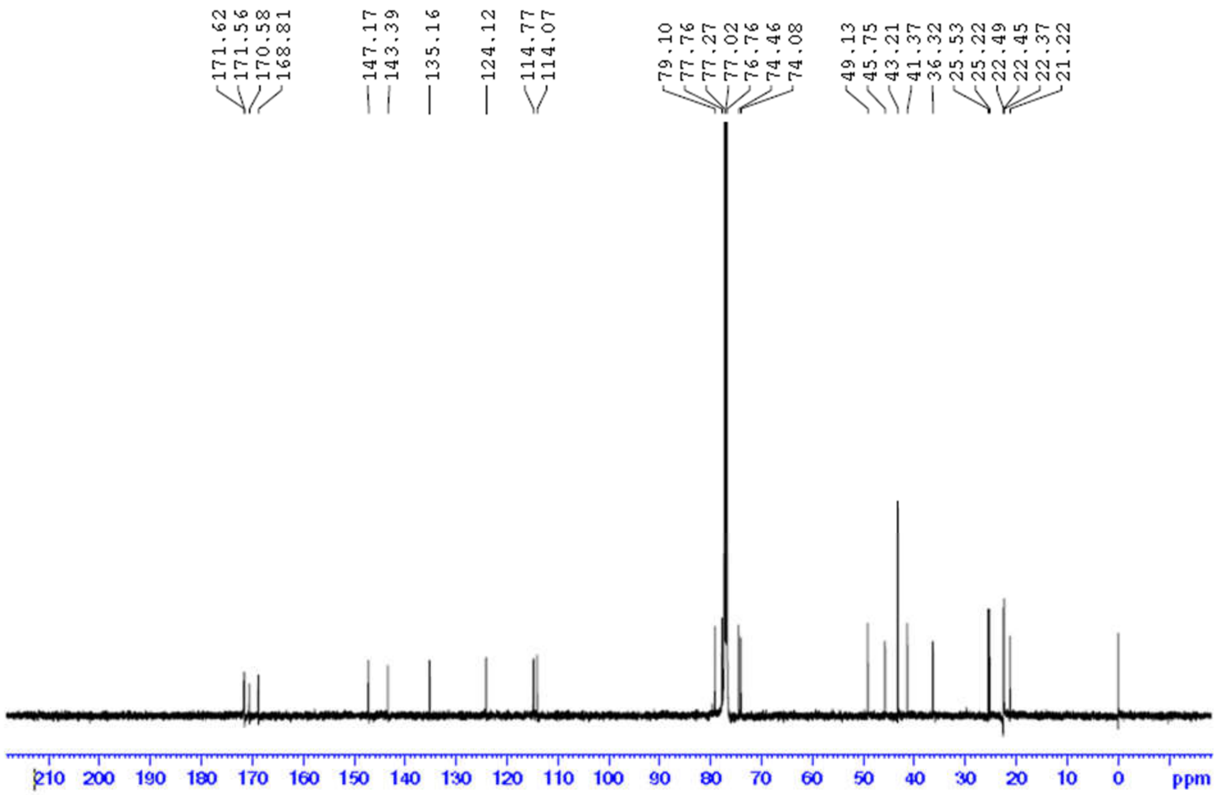

Figure S17: ^{13}C NMR spectrum of compound **3** at 20 K ($CDCl_3$, 500 MHz).

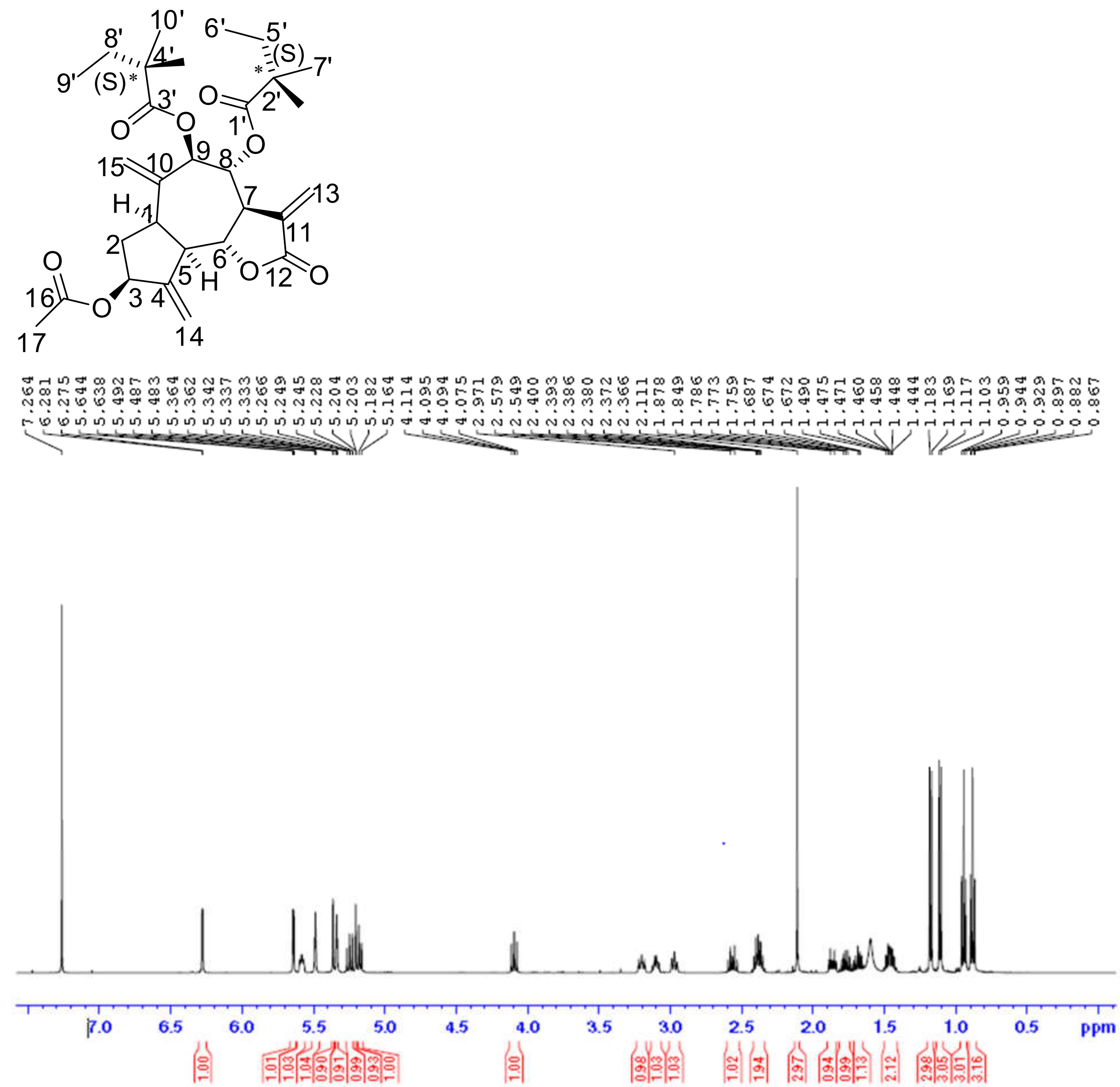

Figure S18: ^{1}H NMR spectrum of compound **4** ($CDCl_3$, 500 MHz).

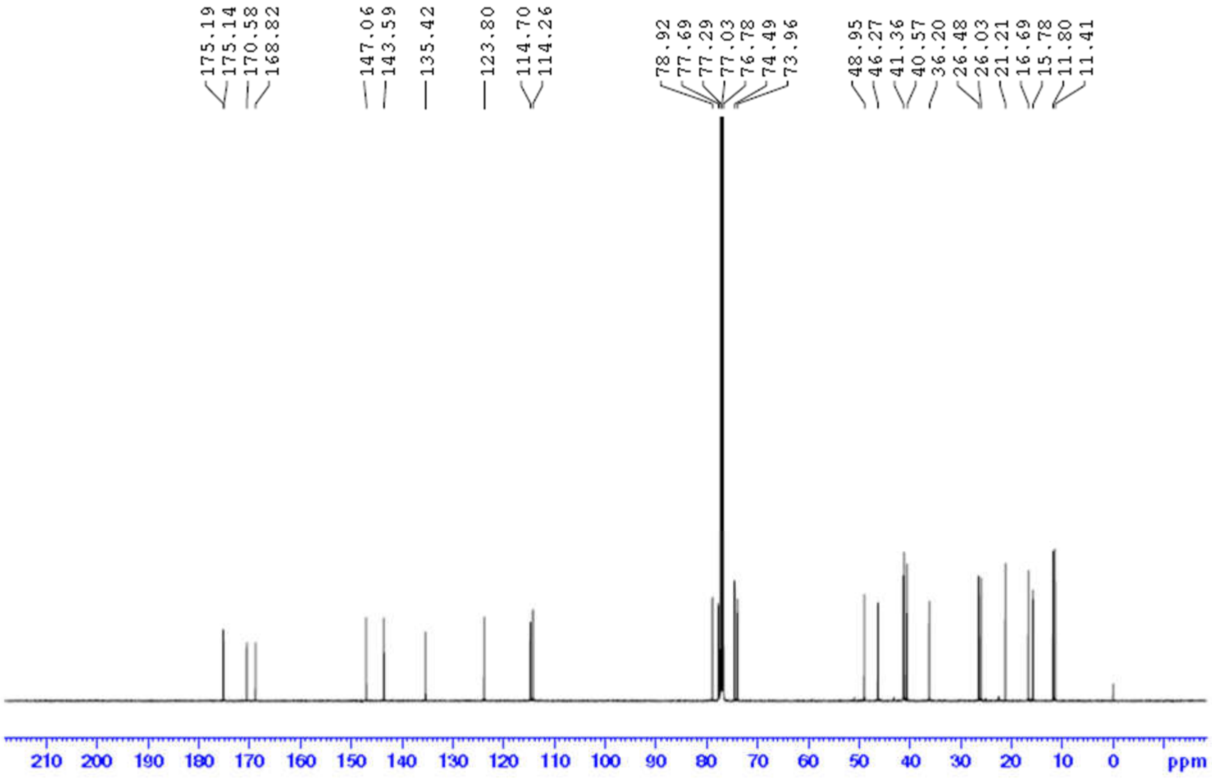

Figure S19: ^{13}C NMR spectrum of compound **4** at 20 K ($CDCl_3$, 500 MHz).

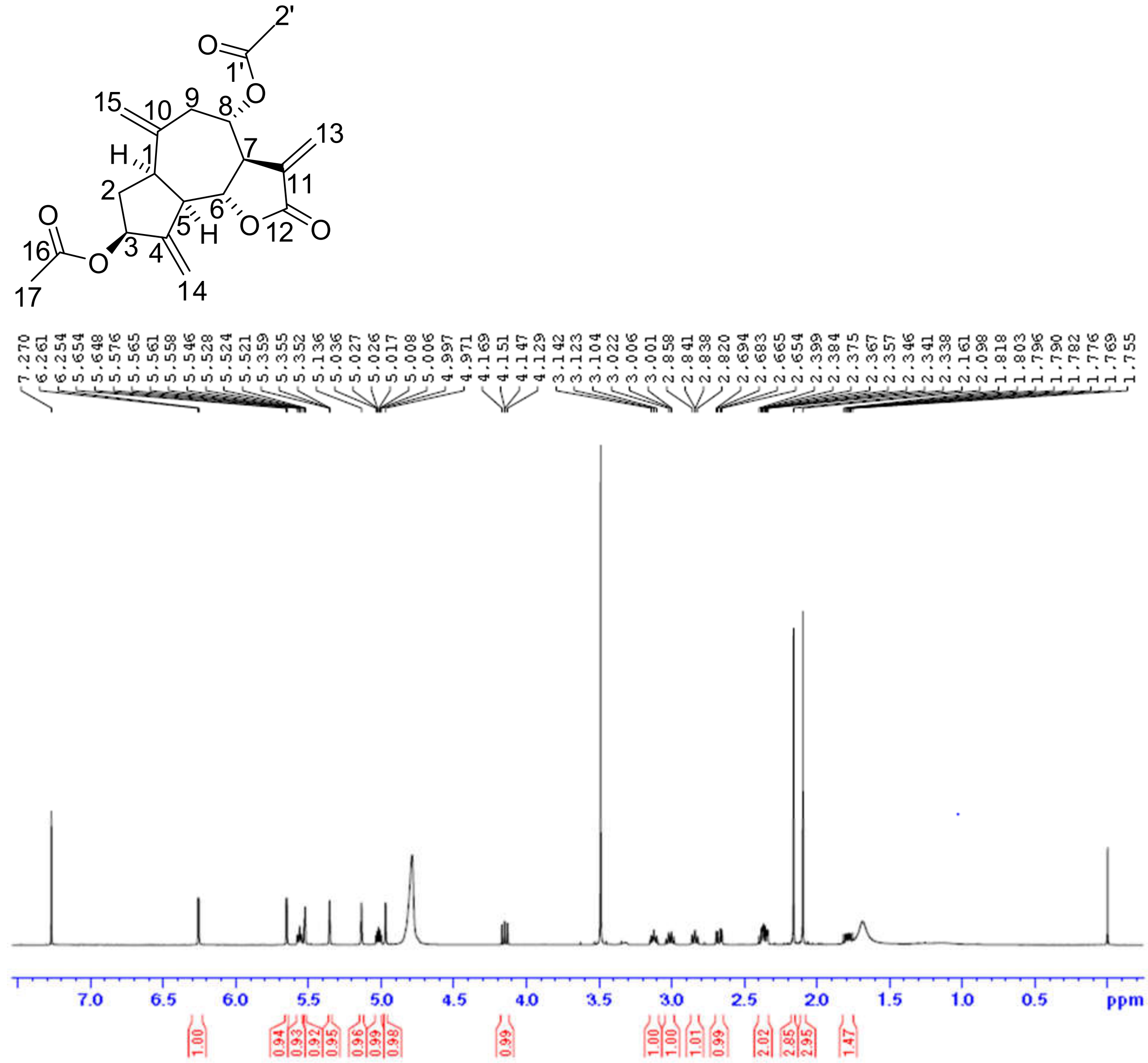

Figure S20: ^{1}H NMR spectrum of compound **5** ($CDCl_3$, 500 MHz).

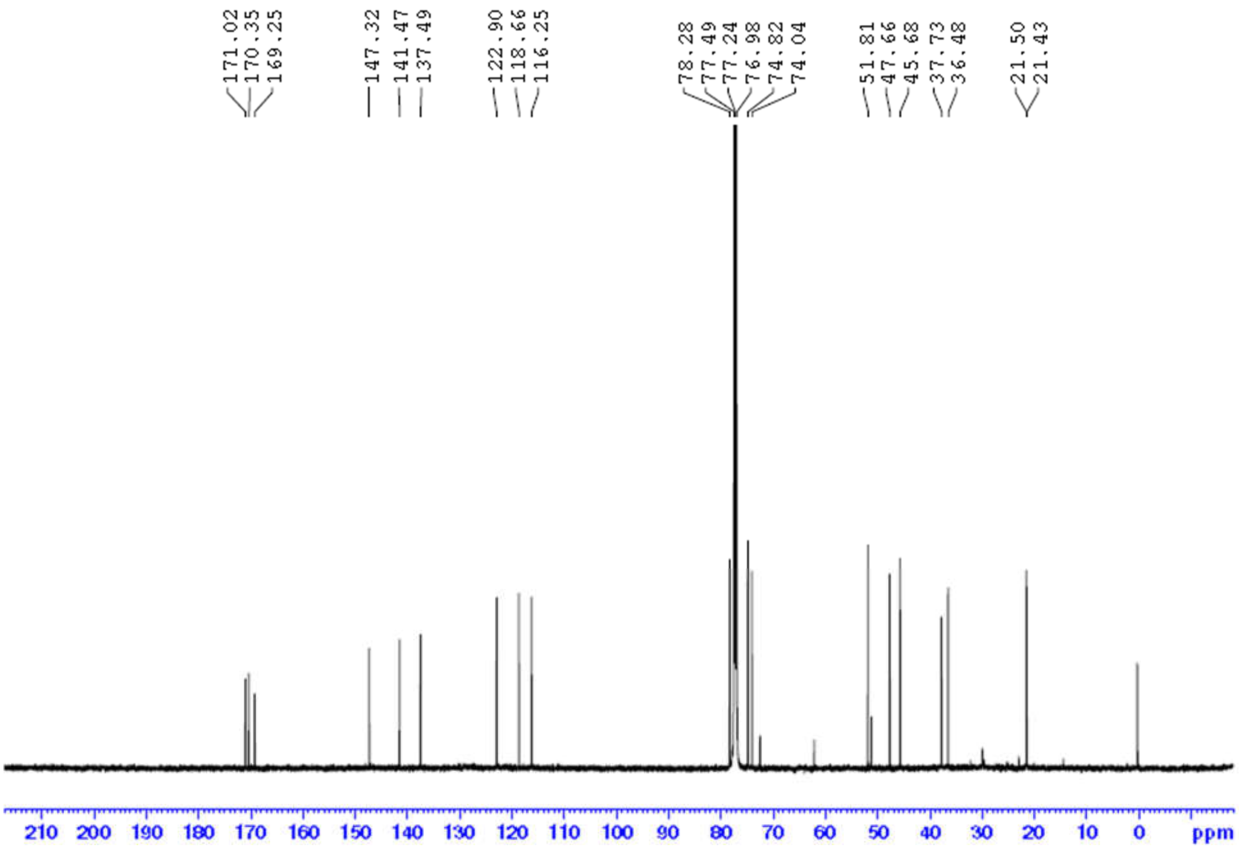

Figure S21: ^{13}C NMR spectrum of compound **5** at 20 K ($CDCl_3$, 500 MHz).

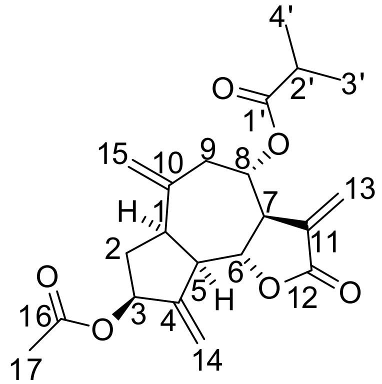

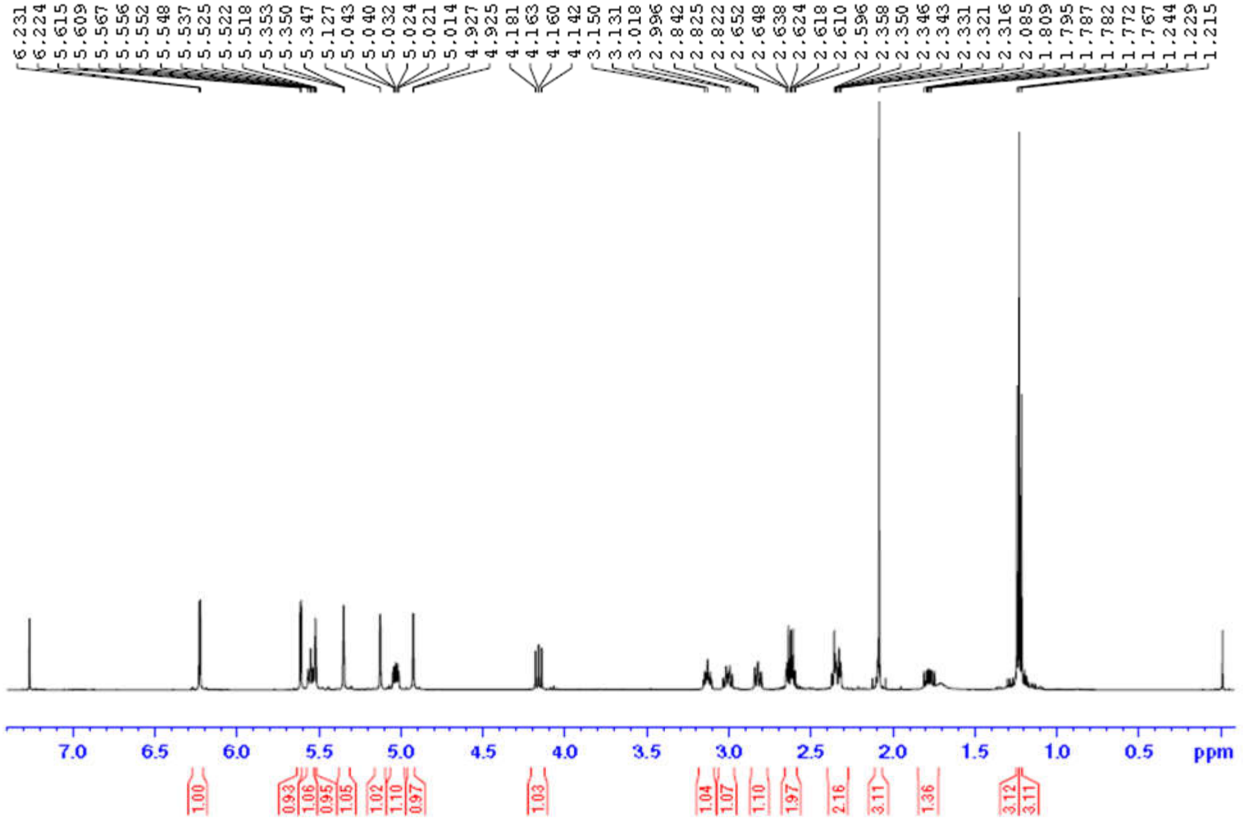

Figure S22: ^{1}H NMR spectrum of compound **6** ($CDCl_3$, 500 MHz).

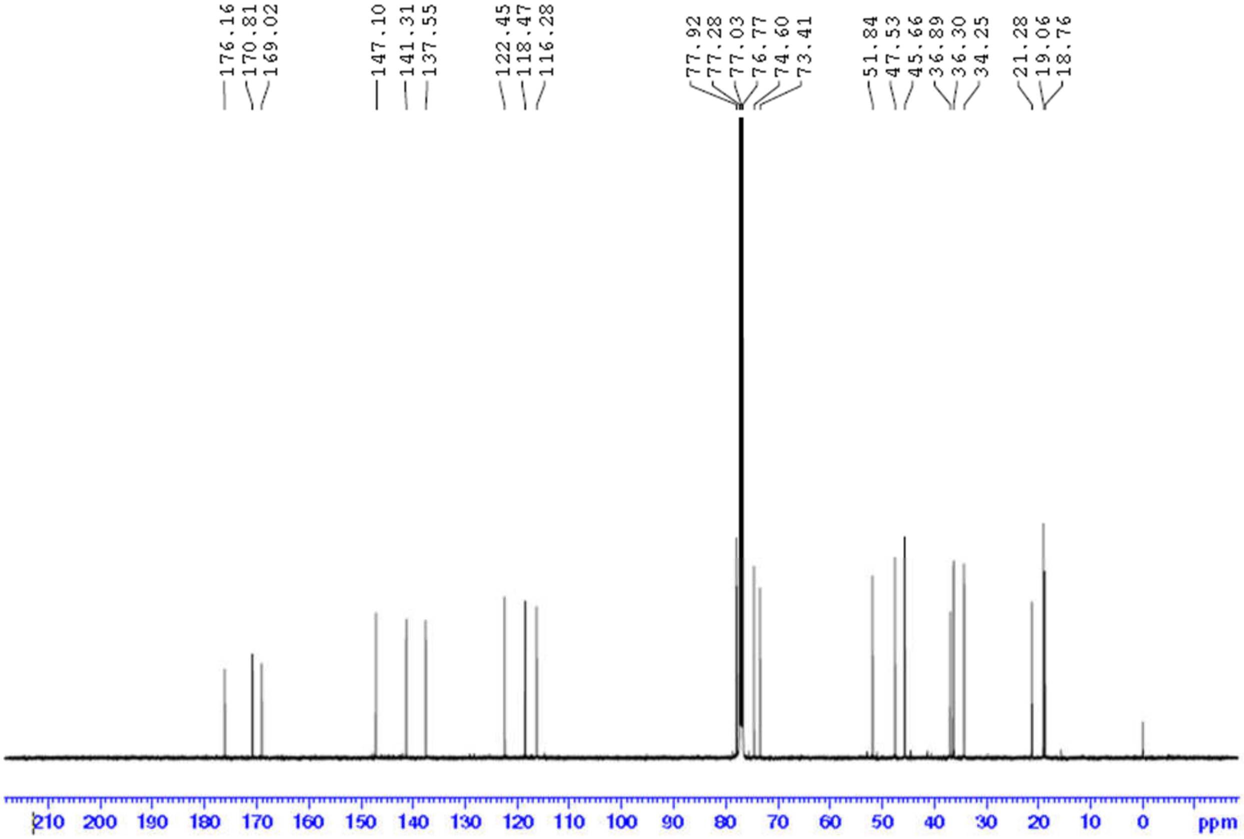

Figure S23: ^{13}C NMR spectrum of compound **6** at 20 K ($CDCl_3$, 500 MHz).

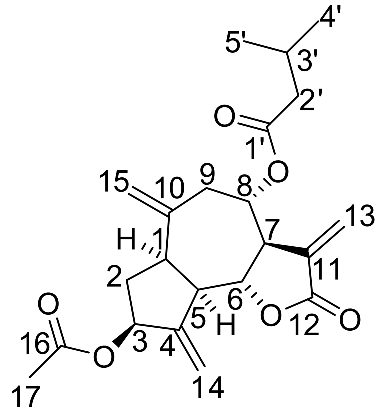

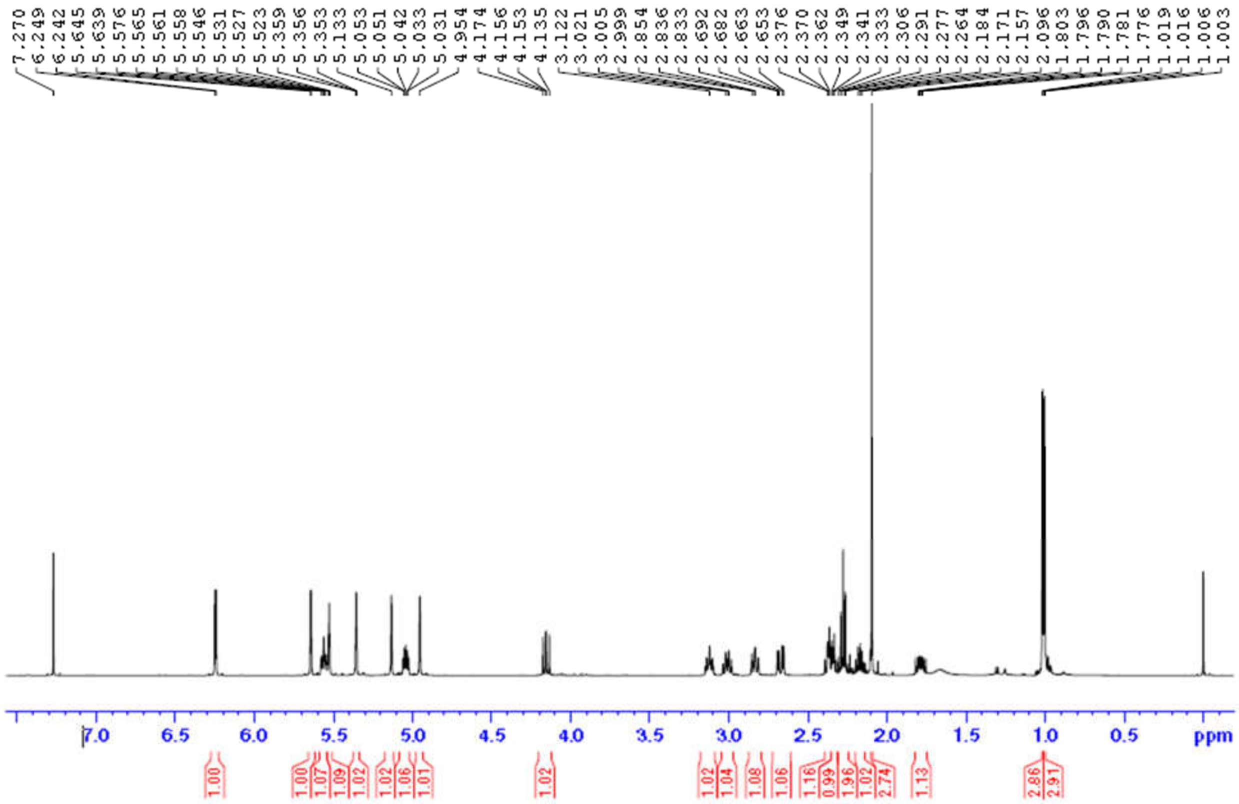

Figure S24: ^{1}H NMR spectrum of compound **7** ($CDCl_3$, 500 MHz).

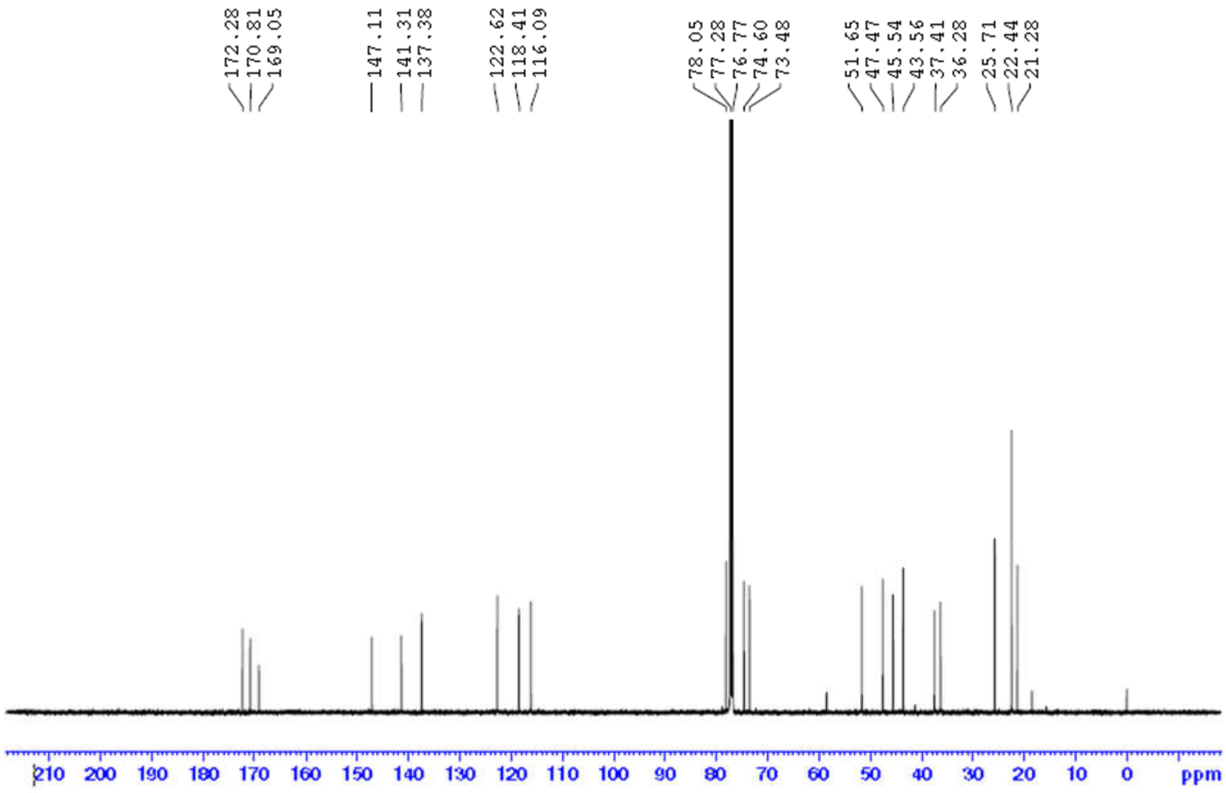

Figure S25: ^{13}C NMR spectrum of compound **7** at 20 K ($CDCl_3$, 500 MHz).

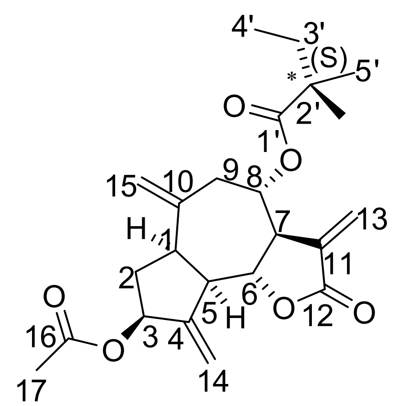

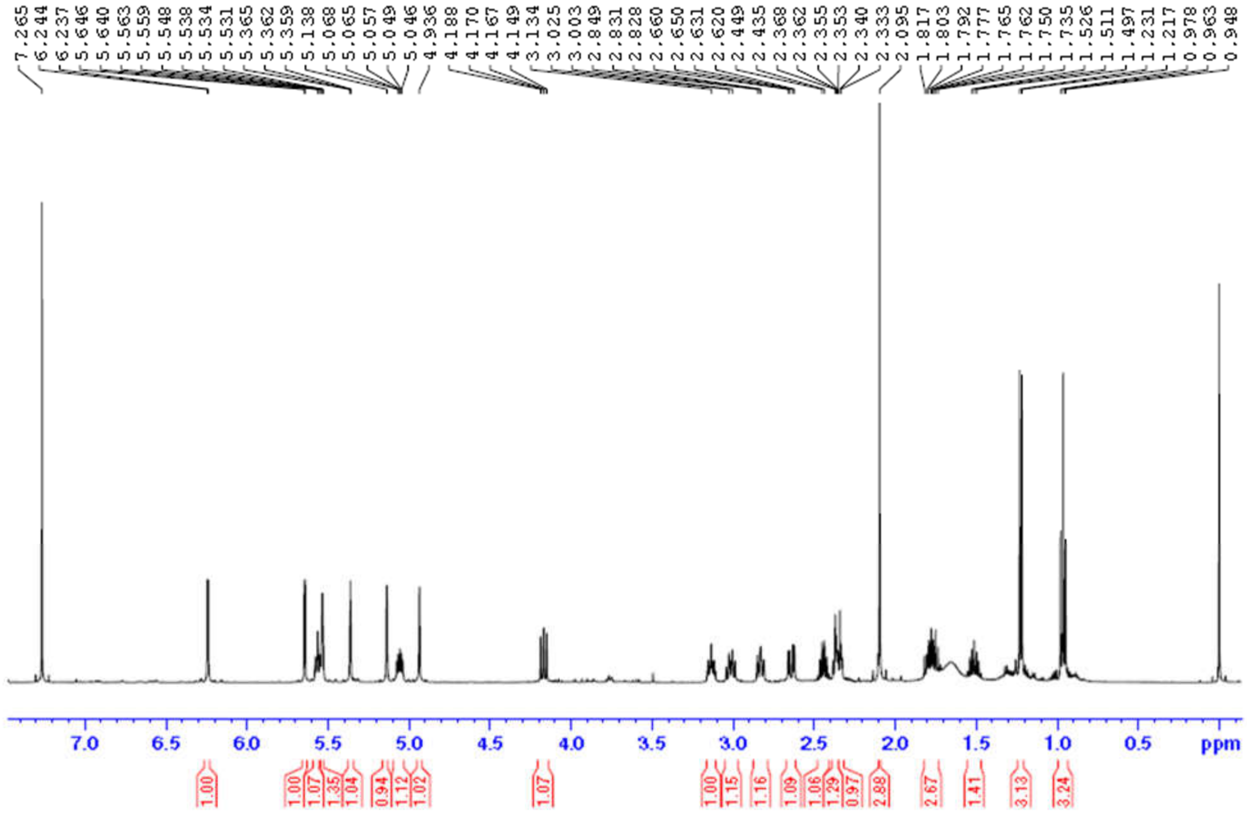

Figure S26: ^{1}H NMR spectrum of compound **8** ($CDCl_3$, 500 MHz).
